# The Effects of Family-Based Programs on Preschool Children’s Screen Time: A Systematic Review

**DOI:** 10.3390/children13040446

**Published:** 2026-03-25

**Authors:** Idurre Arizmendi Sueiro, Markel Rico-González

**Affiliations:** Department of Didactics of Music, Plastic and Body Expression, University of the Basque Country (EHU), 48940 Leioa, Spain; iarizmendi008@ikasle.ehu.eus

**Keywords:** children, mental health, education, family, well-being, television

## Abstract

**Background:** The impact of screen time is having serious adverse effects on people’s lives. Unfortunately, early childhood is the most vulnerable stage in the lifespan, and most children are using television, computers, parents’ and mothers’ mobile phones, or tablets, for longer than recommended. For this reason, the interest of the education community in proposing programs for reducing screen time has grown, which could be of interest for families and professionals in early childhood development and care for children adhering to a healthy lifestyle. For this reason, the objective of this study is to compile programs including families that have tried to reduce preschool-aged children’s time in front of screens. **Method:** The search strategy is designed based on the PICOS framework. A review was conducted in three databases (PubMed, Web of Science, and ProQuest Central) on 11 October 2024, following the PRISMA guidelines. The systematic review is registered in PROSPERO. **Results:** A total of 287 articles were initially found, and 15 met all inclusion criteria. **Conclusions:** The results reveal that programs based on training parents in addition to performing games with children have positive effects for reducing screen time in children up to six years old, even in a specific population.

## 1. Introduction

In early childhood, screen time (ST) has become one of the unhealthy habits that the education community has highlighted as a challenge. ST refers to the amount of time that a person spends in front of a device with a screen, such as a television (TV), computer, tablet, or smartphone [1]. Unfortunately, children’s daily exposure to screen-based gadgets has increased over the past 20 years, although their age of first exposure has decreased, leading researchers to measure ST from the initial years of a person’s life [2]. To date, more than 44% of children under five years of age use smartphones or tablets daily, with the use of each device lasting an average of 30 min [3]. Moreover, a survey reported that 100% of children under four years old use an electronic device (ED), highlighting TV, smartphones, and tablets as the most common. Likewise, the National Health and Nutrition Examination, according to survey data, showed that children up to six years old spend an average of 4 h per day in front of a screen, reducing the possibility of being engaged in non-sedentary habits [4].

In this sense, science has shown the risks of ST on children’s development. These trends have generated concern among parents and health professionals, especially when the exposure time exceeds the established recommendations [5]. To understand how much PA preschool-aged children perform, the World Health Organization (WHO) [6] and various national health entities have established guidelines outlining the minimum physical activity (PA) levels recommended for children in this age group. These recommendations have been implemented in countries like the United Kingdom [7], the United States [8], Australia [9], Canada [10] and Spain [11]. These guidelines have even considered a “best day” for kids’ screen time, sleep, PA, and sedentary behavior [12,13,14]. Adherence to these guidelines indicates better health outcomes and foundational developmental achievements [15]. Health recommendations for the promotion of PA in the kindergarten context could guide society, in general, and families and teachers, in particular, to create environments that promote active lifestyles from early childhood.

When these guidelines are not followed, one of the reported problems is greater sedentary behavior (SB), which affects physical health [16]. As an example, children who spend two or more hours per day in front of a screen significantly increase their sedentary time [17]. In fact, larger ST is directly related to children’s SB, leading to a decrease in PA and greater obesity rates [18]. Unfortunately, these increased obesity rates negatively affect young children’s cardiovascular and metabolic health, as well as their motor development and sleep quality [19,20]. Further, sleeping less than 9.15 h per night is also associated with an increased risk of behavioral problems [21].

But there are more problems, such as the negative effects on children’s mental health and emotional well-being, as well as cognitive development and the development of social competence [22,23]. Going into detail, it has been highlighted that the total ST has been associated with mental health problems [22]. In this sense, a narrative review has reported that several studies showed the relationship between excessive screen use and mental health problems in children and adolescents [24]. Among them, depressive symptoms, anxiety, attention problems, and hyperactivity are the most common, which are associated with lower self-esteem and poorer perceived quality of life [20,25]. Regarding social development, science has highlighted that children who spend more time in front of screens will have fewer face-to-face interactions with others, which limits their ability to develop basic social skills [26]. In addition, children who spend two hours or more per day in front of screens are at increased risk for emotional difficulties, behavioral problems, hyperactivity, peer problems, and prosocial problems. Further, greater exposure to screens has been associated with lower vocabulary, communication, and educational skills, as well as greater problems in peer relationships in children between four years old and the end of early childhood. These effects persisted even after adjusting for socio-familial factors, and it has been shown that spending more than 1.5 h a day in front of screens at age 2 was associated with below-average social skills at age 4.5 [26]. The scientific literature has continued highlighting negative factors associated with other factors, such as nutritional habits. In fact, frequent screen use during meals was linked to unhealthy dietary patterns [27,28]. These issues suggest the need for intervention programs. In fact, a meta-analysis comprised 41 trials with 14,514 individuals. In this meta-analysis, when comparing ST treatments to controls, the overall summary of random effects revealed a slight, positive effect (SMD = 0.26, 95% CI: 0.12 to 0.39) on ST [29].

In this atmosphere, education plays an important role in preventing and mitigating the harmful effects of ST in children during the initial years of their lifespan [30]. In fact, even though ST is typically linked to worse socio-emotional functions, executive functions, language, cognitive, and motor development, its benefits were noted under specific circumstances involving parents: (1) setting a time limit on ST use, (2) parental co-viewing, and (3) exposure to educational content [31]. For this reason, parental intervention strategies to reduce screen time among preschool-aged children have become an important challenge [32]. In this regard, the concept of digital parenting, which describes a collection of techniques parents employ to direct, monitor, and encourage their kids’ usage of digital devices, is highly relevant [33]. In this sense, several systematic reviews have demonstrated the positive effects of adequate parental mediation on the cognitive, social, and emotional development of preschool-aged children. For example, a recent meta-analytical comparison highlighted that the strategy of discussing the content when parents use digital devices with their children is a more effective strategy than unsupervised use [34]. In this way, other systematic reviews on parental mediation, such as Giovanelli et al. [35], show that both restrictive and active mediation techniques—such as setting rules and time or content limits—act as safeguards against the possible dangers of the digital environment and encourage a more balanced and instructive use of technology. In general, the literature about digital parenting indicates that it can positively impact preschoolers’ cognitive development, early literacy, self-regulation, and socioemotional skills when it is based on active engagement, ST regulation, and age-appropriate content selection [33,36,37,38]. This shows that the impact of technology largely depends on how parents mediate and structure its use within the family context.

For these reasons, which support supervision, schools and kindergartens have started proposing supervised programs. For example, Staiano et al. (2018) [39] reported that schools that provide structured environments that promote physical, cognitive, and social development can affect the formation of healthy habits related to the use of technology. Similarly, another study highlighted that by providing access to structured and enriching activities, educational institutions can mitigate observed disparities in screen use [40]. Moreover, limited and supervised ST seems to result in higher PA and lower SB compared to centers without ST restrictions [39]. In addition, since there are associated factors such as changes in parental interactions or socioeconomic factors [41], educational settings provide equal opportunities for children of different socioeconomic levels and function as a space for balancing inequalities.

In this regard, there is a published systematic review that has analyzed programs in educational settings aimed at reducing preschoolers’ ST [30]. However, the importance of including families could be determinant. In fact, the family is the first agent that will influence child development, leading children to internalize behaviors, values, and their first routines [42]. Maybe for this reason, children are particularly receptive to imitating the actions of their parents and caregivers, highlighting the family example as an essential role in establishing positive habits [43]. Several factors can influence children’s screen habits, such as the establishment of clear rules regarding device use, the role models parents set regarding screen use, and the quality of family interactions [44]. For example, the family’s persistence, affection and patience allow these habits to be reinforced naturally, promoting autonomy, self-esteem and emotional well-being in children [45]. Instead, parents with psychological problems (depression or anxiety) may be associated with increased screen use in children, either due to a lack of supervision or the use of devices as entertainment for children [46]. Further, parents who permit children to have technological devices near their children’s bedrooms and without parental supervision were associated with higher ST [47]. Reviewing more of the literature, another article reported that parents with high levels of literacy tend to establish healthy routines for their children, such as regular meals and sleep schedules, and to promote healthy behaviors [48], while parents from low socioeconomic contexts have reported frequent use of screens as an entertainment tool, affecting children’s cognitive and social development [49]. Similarly, the establishment of family routines and parental involvement in these activities is strongly associated with the formation of these healthy habits in children aged 3 to 6 years [50]. All of these studies highlighted the importance of including family.

Therefore, designing intervention programs where families are involved is crucial for an effective reduction in preschool children’s ST. For this reason, the objective of this study is to compile programs including families who have tried to reduce preschool-aged children’s time in front of screens. This work can be useful for teachers in preschools for reducing ST. In fact, it offers tools that educators can apply both at school and at home and encourages policymakers to establish new methods to mitigate the use of these technological devices. In addition, this systematic review can report ideas to share among parents involved in educational settings.

## 2. Materials and Methods

### 2.1. Experimental Approach to the Problem

The systematic review was conducted following the recommendations for conducting systematic reviews in sport sciences [51] and PRISMA (Preferred Reporting Items for Systematic Reviews and Meta-Analyses) guidelines [52]. This systematic review is registered in PROSPERO (CRD420261335850).

### 2.2. Information Sources

To find publications published before 11 October 2024, a thorough search of the three primary databases—PubMed, ProQuest Central (which includes several databases such as ERIC), and Web of Sciences—was conducted.

### 2.3. Search Strategy

The question was stated explicitly using the PICO (Patient, Problem, or Population—Intervention or Exposure—Comparison, Control, or Comparator—Outcome[s]) and the PECOS structure (Population, Exposure, Comparison, Outcomes, and Study Design) designs. The search was restricted to scientific journals and papers whenever feasible (see to exclusion criterion number 6). Journal names and manuscript authors were not hidden from the author.

The aforementioned databases employed the search approach. Every article was downloaded, and each one was examined for eligibility using each inclusion–exclusion criterion individually (see Table 1). When an article met all the inclusion criteria, it was downloaded, included in the review, and its data were extracted to Supplementary Materials S1 and S2. When an article did not meet all the inclusion criteria, it was deleted, highlighting one of the exclusion criteria (applied exclusion criteria). Table 1 shows the search words that were used to find articles that included them in the abstracts and titles of the articles:

(preschool* OR kindergarten OR toddler OR childhood) AND (“screen” OR television OR TV OR computer OR videogame OR tablet OR “mobile phone”) AND (intervention OR program* OR strategy* OR associate* OR correlation OR relation*) AND (reduct*) AND (parent*)

**Table 1:** Inclusion and exclusion criteria for study selection.

No.	Item	Inclusion Criteria	Exclusion Criteria	Search Coherence
1	Population	Children up to six years old	Children more than six years old	(preschool* OR kindergarten OR toddler OR childhood)
2	Intervention/Exposure	Studies or programs involving parents and partnership studies, including parents	Studies or programs not involving parents and partnership studies, not including parents	(“screen” OR television OR TV OR computer OR videogame OR tablet OR “mobile phone”) AND (intervention OR program* OR strategy* OR associate* OR correlation OR relation*) AND parent*
3	Comparison	Any comparison is valid.	-	
4	Outcome(s)	Results related to screens that derive from program effects.	Results that do not derive from program effects.	Reduct*
5	Study Design	Research conducted with at least two groups (it can be a control group or not)	Research conducted with only a group	
6	Other Criteria	Peer-reviewed, original, full-text studies written in English or Spanish.	Written in another language or non-peer-reviewed original full-text studies.	

### 2.4. Eligibility Criteria

To identify information from the articles, one author downloaded the information (title, authors, date, and database) and transferred it into an Excel spreadsheet (Microsoft Corporation, Redmond, WA, USA), where duplicates were removed. The remaining articles were screened by two authors to select those articles that met all inclusion criteria (Table 1). In the process, if these two authors could not agree on whether an article should be included/excluded, a third author (without authorship in this systematic review) helped us make a decision.

### 2.5. Data Extraction

In compliance with the Cochrane Consumers and Communication Review Group’s data extraction template, data extraction was prepared using an Excel spreadsheet. The spreadsheet was used to assess inclusion and exclusion requirements for all selected studies. Reasons for exclusion were noted for full-text articles that were not included in the analysis. All records were stored in the spreadsheet. It was made by an author, but a second author was consulted if there was any uncertainty. Once all records were selected and downloaded, the information of each of them was extracted.

### 2.6. Quality of Studies

Additionally, the second version of the Cochrane risk-of-bias tool for randomized trials was used (RoB 2). The authors assessed different key domains: random sequence generation (1 item), allocation concealment (item 2), blinding of participants and personnel (item 3), blinding of outcome assessment (item 4), incomplete outcome data (item 5), selective reporting (item 6), and other bias (item 7) [53]. Each domain was evaluated and classified using low risk of bias (+), high risk of bias (−), or some concerns or unclear risk of bias (?). Two authors conducted RoB 2.0 assessments (I. A. S. and M.R.-G.), with disagreements resolved through discussion.

## 3. Results

### 3.1. Identification and Selection of Studies

**Figure 1:**
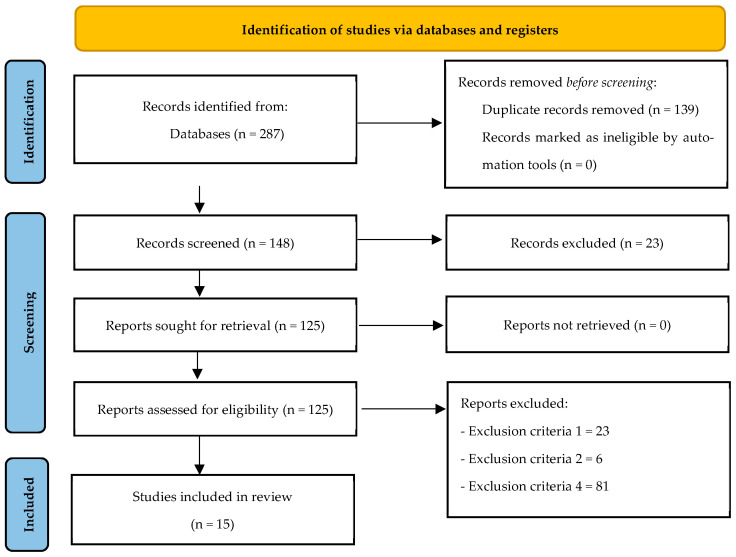
Flow diagram of the study.

A total of 287 original articles were found (Web of Science: 122; PubMed: 88; Proquest Central: 74; external source: 3), of which 139 were duplicates. Therefore, 148 articles were identified. After reviewing the titles and abstracts, 23 articles were excluded because they did not meet the fifth inclusion criterion. The remaining 125 articles were reviewed in their entirety; 23 articles were excluded for not meeting the first inclusion criterion, 6 articles for not meeting the second inclusion criterion, and 81 articles for not meeting the fourth inclusion criterion. Thus, 15 articles met all inclusion criteria and were included in the final qualitative synthesis (see Figure 1).

### 3.2. Quality Assessment

According to the RoB-2 scale, the study shows that the included studies assume risk of bias in several areas, primarily in allocation concealment (Risk of bias = low: 7; moderate: 1; high: 7) and in both blinding-related items: blinding of people and participants and blinding of outcome assessment. In addition, most of the articles showed the dropout of participants because some participants completed a pretest but not a posttest assessment. However, this concern is usual in interventions done in schools. In fact, some of the articles declared that it was not possible due to the nature of the intervention. Despite this, most of the studies satisfy the first inclusion criterion. Lastly, all studies were categorized as having some concerns in the elements relating to “other bias” because several difficulties could present a danger of bias. Table 2 displays the findings of the assessed risk of bias (Table 2).

### 3.3. Study Characteristics

In total, 13 intervention programs out of 15 showed a reduction in children’s ST, while Tuominen et al. [64] and Birken et al. [67] reported non-significant effects. These 15 studies were classified into three different groups, considering the type of program and target population: (1) programs focused on parental training, (2) programs based on parental training and alternative activities with children, and (3) programs targeting a specific population. The table about characteristics of included studies (Supplementary Materials S1) and the table about the characteristics of each intervention program (Supplementary Materials S2) are included as Supplementary Materials. The names of the programs, duration, and their effect sizes and significance were analyzed.

#### 3.3.1. Programs Focused on Parental Training

There are six studies aimed at reducing ST through parents’ formation. These programs are: PSTRPP [54], parent-focused intervention [55], Family@play [56], Case manager support [57], intervention for reducing *ST* [1], and parental education for limiting *ST* in early childhood, targeting children under one year of age [62]. The duration of these programs lasted two weeks [54], six months [55,62], two years [1,56], and three years [57].

Looking into ST reductions, effect size (ES) and significance, most of the programs score significant effects, even the program with three sessions (3 h each) over two weeks, which reduced ST from 127.14 to 70.64 min/day (57 min/day) with a regression coefficient of −49.36 ± 9.55 (95%CI: from −68.07 to −30.64; *p* < 0.001) [54].

Regarding the programs of six months, both scored significant differences between pre- and post-test. First, Feng et al. [55] found differences in both programs in two measures (post-test and follow-up): the integrated-focus group (33 min/day of reduction; ES = post-test: small-moderate (d = 0.42); follow-up: small (d = 0.27)) and dyadic-focus group (31 min/day of reduction; ES = post-test: moderate-large (d = 0.66); follow-up: large (d = 0.75))

The program lasted two years, and Hinkley et al. [56] found greater decreases in total ST use among children in the INT (adjusted difference [95% CI] = −31.2 min/day [−71.0–8.6] Cohen’s d = 0.70) when compared with children in CON.

Finally, when children were involved in a program for three years in a third study, the study showed a significant reduction by 37 min/day in total viewing time (95% CI: 5.6–68.7), including a marginally significant reduction by 29 min/day in viewing of commercial content (95% CI: −4.6–63) [57].

But these results, scored in studies involving preschool children, remain even with children who are less than one year old. In fact, Poonia et al. [62] found that children in the INT had ST > 1 h/day as compared to 53% (32/60) (*p* < 0.001) in the CON. Median (IQR) for total screen duration in the educational group was 35 min (30, 49) min/day compared to 75 (50, 90) min/day in the CON (*p* < 0.001).

In general, studies achieved a reduction of 29–57 min/day after interventions of at least two weeks. In addition, studies that analyzed follow-up found significant differences [54,55], such as a large ES (d = 0.75), in the case of Feng et al. [55].

#### 3.3.2. Programs Based on Parental Training and Alternative Activities with Children

Seven intervention programs aimed at reducing ST through both strategies simultaneously: parents’ training and alternative activities with children. These programs are different because some of them are designed to be carried out at home (Stop and Play [60] “movement-to-music video program” [64], an intervention modeled around previously published screen-time interventions [67]) and others to be carried out at school (PLUMS [59], F5K TV reduction programs [58] “Intervention to reduce children’s television viewing” [66] and behavioral interventions to improve healthy habits [65]). Due to the different nature of each intervention, programs are detailed in Supplementary S2 and discussed in the discussion. These programs lasted three months [67], eight months [59], ten months [60,66], and two years [58,64,65].

Most of the programs (five out of seven) found significant differences after the intervention regarding the ST reduction. First. Kaur et al. [59] found a significant difference (*p* < 0.05) of 27.7 min/day between INT (102.6 ± 98.5 min) and CON (130.3 ± 112.8 min) groups [95% CI 5.1, 50.3] at the post-intervention (post-test eight months after) assessment. In addition, a significant reduction in ST (β = −35.81 min, CI −70.6, −1.04) from baseline (β = 123.1 min) to the follow-up phase (β = 116 min) was found. The results of eight months of intervention remain when the program lasted 10 months. First, Raj et al. [60] highlighted that INT showed significantly reduced children’s ST compared with the CON (β = −202.29, 95% CI −224.48 to −180.10; *p* < 0.001). Second, Dennison et al. [66] showed that before the intervention, the INT and CON viewed 11.9 and 14.0 h/wk of TV/ videos, respectively. Afterward, children in the intervention group decreased their TV/video viewing by 3.1 h/wk, whereas children in the CON increased their viewing by 1.6 h/wk, for an adjusted difference between the groups of −4.7 h/wk (95% CI, −8.4 to −1.0 h/wk; *p* = 0.02). Finally, these results remain when the program lasted two years. First, Romo and Abril-Ulloa [65] found additional beneficial effects of the enhanced intervention that were not observed with the Pilot Intervention, including a reduction in excessive weekend ST (−7.6%, *p* = 0.03). Second, in the intervention by Mendoza et al. [58] children decreased from 76.2 (9.9) at Time 1 to 52.1 (10.0) at Time 2, whereas control children remained about the same from 84.2 (10.5) at Time 1 to 85.4 (10.5) at Time 2. The relative difference from Time 1 to Time 2 was −25.3 (95% CI = −45.2, −5.4) min for INT vs. CON children (N = 160, *p* = 0.01).

However, some studies highlighted non-significant differences between groups. First, Tuominen et al. [64] highlighted that among the children in the CON, ST increased from 89 (SD 37) to 99 (SD 41) min/d, but no statistically significant differences between groups were found in primary or secondary outcomes. Second, Birken et al. [67] found no significant differences in mean total weekday minutes of ST (60, interquartile range [IQR]: 35–120 vs. 65, IQR: 35–120; *p* = 0.68) or mean total weekend day minutes of ST (80, IQR: 45–130 vs. 90, IQR: 60–120; *p* = 0.33) between the intervention and control group.

#### 3.3.3. Programs Targeting a Specific Population

There are two studies aimed at reducing ST in the population with a specific population: children with neurodevelopmental disorders [61] and children with elevated or rapidly increasing body mass index [63]. The duration of the interventions lasted from six months [63] to three years [61]. Going into detail, for the study by Bahadur et al. [61], the baseline median screen time before the intervention was 5.0 h a day for all participants (Interquartile range (IQR): 4–9); after the intervention, the median screen time for all participants decreased to 2.0 h a day (IQR:1–3) (*p* < 0.001). Before the intervention, the median percentage spent co-viewing was described as 12.5% of screen time, compared to 40% of screen time after the intervention (*p* = 0.007) for all participants. In the second study, Tucker et al. [63] FNPA scores improved in treatment vs. control (4.6 ± 4.6 vs. 0.1 ± 4.2; *p* < 0.001), and ST (h/day) decreased (−0.9 ± 1.8 vs. 0.3 ± 1.1; *p* < 0.001).

## 4. Discussion

The objective of this study is to compile programs, including families, that have tried to reduce preschool-aged children’s time in front of screens.

### 4.1. Studies with Programs Based on Parent Training

One of the main types of intervention to reduce ST in preschool children is aimed at training parents through different strategies. The interest in this method may be because the family is the first social environment of the child, and it is essential that parents are trained to set limits and encourage healthy use of screens in the family environment [68]. These programs are PSTRPP [54], parent-focused intervention [55], Family@play [56], Case manager support [57], intervention for reducing ST [1], and parental education for limiting ST in early childhood, targeting children under one year of age [62].

First, Boonmun et al. [54] evaluated the effects of a program called PSTRPP on the reduction in children’s ST. For this purpose, they divided 67 families with children aged two to five years into a control group (CON) (n = 32) and an intervention group (INT) (n = 35). The members of the INT conducted three sessions (instructional session, restrictive session, and parent–child interaction session) with the aim of providing parents with strategies on how to reduce children’s ST. The results of the study showed that the program reduced children’s ST both in the short term, one week, and in the follow-up, two months. Going into detail, ST was reduced from 127.14 to 70.64 min/day with a regression coefficient of −49.36 ± 9.55 (95%CI: from −68.07 to −30.64; *p* < 0.001) [54]. In a second study, Zimmerman et al. [57] proposed a program to reduce TV time and replace commercial TV with educational content. They divided 67 families into an INT (n = 34) and a CON (n = 33). In the intervention, parents were advised to reduce their children’s ST to one hour or less per day and were provided with educational media as an alternative to commercials. The results obtained state that compared to the CON, families assigned to the INT experienced a significant reduction of 37 min/day in total viewing time (95% Cl: 5.6–68.7), including a marginally significant reduction of 29 min/day in commercial content viewing (95% Cl: −4.6–63) of 29 min/day in commercial content viewing (95% Cl: −4.6–63). Third, Yilmaz et al. [1] evaluated, in the primary care setting, an intervention divided into INT (n = 187) and CON (n = 176), where materials were delivered to 363 families. Families in the INT were asked to read books daily with the children; they were offered alternatives to ST and were encouraged to remove the TV from the children’s room. In addition, they were given a screen-free home assessment to raise awareness. The results obtained indicate that parents in the INT group reported spending less time in front of a screen and exhibited less aggressive behavior than those in the CON group. Third, Feng et al. [55] evaluated a program involving a total of 147 parent–child pairs divided into three groups: two INT groups and one CON group: the integrated approach group (n = 49), the dyadic approach group (n = 47), and the CON group (n = 51). The integrated approach group, in addition to receiving online training workshops, received individual reports on their children’s current behaviors, deficiencies, and recommendations for improving behaviors and habit development. However, the dyadic approach group received the same intervention contents as the integrated approach, with the exception that the intervention materials were limited to PA and SB (including sedentary ST). The results indicated that preschoolers in both INTs reduced ST after the intervention and at follow-up: the integrated-focus group (33 min/day of reduction; ES = post-test: small-moderate (d = 0.42); follow-up: small (d = 0.27)) and the dyadic-focus group (31 min/day of reduction; ES = post-test: moderate-large (d = 0.66); follow-up: large (d = 0.75)). Thus, they concluded that both intervention approaches were effective in alleviating the compositional decline in ST reduction and revealed a possible greater efficacy of the integrated approach in promoting general movement behaviors among preschoolers.

However, given that these results have shown the viability of the use of programs in children up to five years of age, it would be interesting to evaluate whether, specifically in the population under three years of age, the use of these programs is also effective in reducing ST. In this regard, Hinkley et al. [56] evaluated the effects of the Family@play program by dividing 22 families into an INT group (n = 12) and a CON group (n = 10). As in the previous study, this intervention was based on increasing parents’ knowledge and imparting recommendations and applications of strategies to reduce TS in children. The results indicated that, compared with children in the CON group, there was a greater decrease in total ST use among children in the INT group (adjusted difference [95% CI] = −31.2 min/day [−71.0–8.6] Cohen’s d = 0.70). Statistical differences for other outcomes were from moderate to large for individual ED (e.g., TV viewing, DVD/video viewing). Thus, the Family@play program proved to be a feasible and acceptable intervention for families of 2–3-year-olds. Further, Poonia et al. [62] evaluated the impact of a program on children under one year old. To do so, they examined 120 healthy children aged 9–10 months by dividing them into a CON group (n = 60) and an INT group (n = 60). Parents in the INT received an educational talk to incorporate parenting skills, increase play with the children and modify adult media habits. The results showed that the mean total ST in the educational group was 35 min/day compared to 75 min/day in the CON group (*p* < 0.001): INT had ST > 1 h/day as compared to 53% (32/60) (*p* < 0.001) in the CON. Median (IQR) for total screen duration in the educational group was 35 (30, 49) min/day compared to 75 (50, 90) min/day in the CON (*p* < 0.001). Thus, parental education from the first year of life is a promising intervention to reduce children’s exposure to screens.

In conclusion, programs that exclusively focus on parents’ training have been shown to be effective in reducing ST (29–57 min/day after, at least, two weeks of parents’ training-based interventions) in children under five years of age, even with large effects in the long-term (d = 0.75). [55]. It is very interesting because even interventions with low intensity/duration (three sessions (3 h each) over two weeks) showed a reduction of 57 min/day of ST (regression coefficient: −49.36 ± 9.55 (95%CI: from −68.07 to −30.64; *p* < 0.001)) [54]. Through sessions, educational materials and counseling, it has been possible to modify children’s media habits in both the short and medium term. Moreover, it has been shown that these interventions are feasible even at very early ages, such as in children under three years old or even one-year-olds. Therefore, training parents from early stages is a promising strategy to reduce children’s exposure to screens.

From these results, clinical and public health implications can be extracted. In fact, preschool-aged children’s ST reduction is a complicated, multidimensional problem that calls for an all-encompassing strategy involving parents, educators, and legislators [69]. Hence, following the outcomes extracted from this systematic review, strategies such as parents’ ST reduction to one hour or less per day, the use of educational media instead of commercials, or reading books daily instead of investing time in front of screens have been suggested to involve parents in this challenge. Therefore, counseling sessions during well-child visits may be interesting, not only after intervention, but also for the future, as was highlighted by these studies that have analyzed programs’ effects some months after the end of the intervention. These sessions can be done in clinical settings. Therefore, bringing this evidence from the clinical/family setting into the school environment transforms the school into a family training center. The practical implications are that the school not only educates the child, but also “trains” parents to ensure consistency between the classroom and home. In fact, it has been suggested that school interventions that do not actively involve parents have limited success in reducing total ST. The reasons for the positive effects found in the programs included in this systematic review may be due to the mirroring effect, where the child changes when parents change. These measures could be considered and lead pediatricians, parents, and teachers to a reduction in children’s ST, and subsequently, better cognitive, socioemotional, and motor development, even mitigating mental health problems.

From a critical point of view, the reader should consider these results with caution. Although the results seem positive, the risk of bias is derived from the fact that the sample was not randomly selected, nor was allocation concealment done in one study [54], as well as the declared non-possibility of blind participants, personnel, and outcome assessment in most of the other studies, suggest that these outcomes should be interpreted with caution, awaiting future blinded randomized controlled trials that reinforce these findings.

### 4.2. Research Based on Preparing Adults and Proposing Games with Children

In addition to parent training, including activities with children to reduce infant ST has also been investigated. These programs have been divided into two sub-sections:

• Programs aimed directly at the home environment: Stop and Play [60], the “movement-to-music video program” [64], and an intervention modeled around previously published screen-time interventions [67].
• Programs in which children’s activities have been carried out at school: PLUMS [59], F5K TV reduction program’s [58], the “Intervention to reduce children’s television viewing” [66], and behavioral interventions to improve healthy habits [65].

#### 4.2.1. Programs Aimed at the Home Environment

Some of the authors have designed programs directly aimed at the home environment. It is interesting because it seems that children at home, instead of those attending daycare, have more ST and SB [64]. First, Raj et al. [60] aimed to develop, implement, and evaluate the effectiveness of a program called Stop and Play, which was implemented for 10 months. The sample was divided into two homogeneous groups (n = 360 pairs). Then, the authors subjected the legal guardians of the INT to talks with professionals aiming to improve parenting skills to reduce the ST of minors, and the children to participate in self-directed games as an alternative to ST. The results of this intervention showed that, even three months after the intervention, the INT showed a significant reduction in child ST compared to the CON group (β = −202.29, 95% CI −224.48 to −180.10; *p* < 0.001).

However, unlike the general trend of finding positive effects on ST reduction, two studies did not show significant differences between groups. In this regard, Tuominen et al. [64] evaluated a program implemented for eight weeks of movement—a music video that could reduce SB and increase PA in mother–child pairs in the home environment. In this study, 203 families were divided into INT (n = 102) and CON (n = 101). The intervention provided the mothers and children in the INT with the DVD of the musical movement video program every other day from the beginning of the second week to the end of the eighth week. The results revealed that no statistically significant differences were found between the groups, nor between the pre- and post-test of the INT. However, the ST of the children in the CON increased from 89 to 99 min/day. In the second study, Birken et al. [67] evaluated the program for three months. They divided 160 families into INT (n = 81) and CON (n = 79). Parents in the INT received 10 min of behavioral counseling by trained study staff directly after the health maintenance visit, in addition to suggestions such as eating meals without the TV on and removing the TV from the child’s room. On the other hand, contingency planning for the time spent without watching TV was promoted. Finally, parents of children in the INT and CON received standardized counseling from trained study personnel on safe media use, including information on TV rating systems, internet safety, and limiting exposure to violent programs. Results showed no significant differences in total weekday median minutes of ST (60, interquartile range [IQR]: 35–120 vs. 65, IQR: 35–120; *p* = 0.68) or total weekend median minutes of ST (80, IQR: 45–130 vs. 90, IQR: 60–120; *p* = 0.33) between the INT and CON groups. Adjusting for baseline BMI, there was a reduction in the number of weekday meals in front of the TV (1.6 ± 1.0 vs. 1.9 ± 1.2; *p* = 0.03) but no difference in the number of TVs in the bedroom.

Therefore, although the effects of programs aimed at the family environment on reducing children’s ST are not clear, training parents with behavioral counseling, improving parenting skills to reduce children’s ST, imparting suggestions (e.g., eating meals without the TV on and removing the TV from the child’s room), and activities such as musical movement videos or self-directed activities with children could avoid increments on children’s ST.

#### 4.2.2. Programs in Which Children’s Activities Have Been Carried at School

On the other hand, those studies that involve the inclusion of activities within schools, as well as with families, are grouped together. These programs may be of special interest to professionals working in schools with children in early childhood education.

In this regard, Kaur et al. [59] evaluated the effectiveness of the PLUMS program over a period of eight months. They divided 340 families into an INT (n = 170) and a CON (n = 170). The INT conducted a program divided into two modules containing thematic activities for children and videos for parents or caregivers to help parents change the home media environment, offer alternatives to ST, provide tips and hints for parenting skills, and involve the child in media-free activities of his or her choice. The results of the study showed that the mean difference in ST on a typical day [27.7 min, 95% CI: 5.1, 50.3], in contrast to the previous assessment, decreased significantly (*p* < 0.05) in the INT group (102.6 + 98.5 min) compared to the CON group (130.3 + 112.8 min). In the statistical analysis, a significant reduction in ST (β = −35.81 min, CI −70.6, −1.04) was observed from the baseline phase (β = 123.1 min) to the follow-up phase (β = 116 min). Thus, the PLUMS program significantly reduced the mean ST of the children on a normal day. In a similar way, Mendoza et al. [58] evaluated the effects of the FSK program, applied over two school years. To do so, they divided 160 families with children aged 3–5 years into INT (n = 90) and CON (n =70). The families in the INT replaced the screens with alternatives by means of educational lessons. In addition, a newsletter was sent to parents with alternatives to ST. The results obtained were that children in the INT decreased from 76.2 min at time 1 (pre-intervention) to 52.1 min at time 2 (immediately after the intervention), while children in the CON remained virtually the same, from 84.2 min at time 1 to 85.4 min at time 2. The relative difference between time 1 and time 2 was −25.3 (95% Cl= −45.2, −5.4) min for children in the INT versus the CON (n = 160, *p* = 0.01), so the F5K program was effective in reducing the time preschoolers spent watching TV by more than 25 min per day. In a third study, Dennison et al. [66] designed a study to evaluate the effects of a program implemented for 39 weeks. The authors divided the sample into INT (n = 93) and CON (n = 83). Participants in the INT conducted seven educational sessions in the classroom and after each session, materials and activities were sent home with each child to encourage parent–child interaction. The results of the intervention revealed that children in the INT reduced their TV/video consumption by 3.1 h/week, while those in the CON increased it by 1.6 h/week, an adjusted difference between the groups of 4.7 h/week (95% confidence interval, −8.4 to −1.0 h/week; *p* = 0.02). Finally, Romo and Abril-Ulloa [65] subjected 307 families to two interventions: group INT 1 (n = 155), called pilot intervention (8 months), and group INT 2 (n = 152), called enhanced intervention (which lasted 6 months). The intervention was based on Social Cognitive Theory and was developed after conducting a series of focus groups with parents, teachers and school administrators. The results showed a reduction in excessive ST during the weekend (−7.6%, *p* = 0.03) in the INT 2 group.

In conclusion, programs that, in addition to training parents to improve the home environment, implemented activities with the children within the schools, showed unanimity in finding a significant reduction of up to 25 min in ST once the programs were carried out, at least as long as these programs lasted eight months or more. These results may be very interesting for professionals working with children in educational settings. However, it should not be overlooked that the combination of parental support seems to be key to achieving sustained and significant reductions in the daily use of screens. Therefore, their integration into primary health care and preschool education programs is recommended.

These conclusions are of interest to the educational community, especially from early childhood, because children are sensitive to neuroplasticity during the initial years of their lives [70]. For this reason, policymakers should consider implementing measures in educational settings. From a clinical and public health implications perspective, the findings from this systematic review strategy, such as improving parenting skills while children participate in self-directed games, suggestting parents to change the home media environment, offer alternatives to ST, provide tips and hints for parenting skills, while involving the child in media-free activities of his or her choice, encourage parents to replace ST with educational lessons or, after educational sessions in the classroom and after each session, send materials and activities to encourage parent–child interaction at home. All these suggestions go together with parents’ formation for reducing children’s ST. From these findings, we encourage schools to involve health centers in these sessions aimed at parents’ formation. In addition, it could be of interest if schools and health centers are encouraged by policy to communicate in both directions. This would allow for the implementation of a shared “Digital Health Record.” During pediatric visits in schools, the pediatrician determines the danger of excessive ST and “prescribes” participation in the school’s workshops. In this manner, the school delivers the report to the pediatrician after certifying attendance at training sessions. The parent feels supported by two distinct authorities as a result of this circle of duty. To this aim, policymakers could fund the opening of school playgrounds and libraries outside of school hours for guided play activities where parents and children can interact without screens. Finally, joint training modules for preschool teachers and primary care pediatricians on behavior modification strategies can be created.

However, as in the other sections, these results should be considered with caution for the following reasons. On the one hand, the studies that encourage children to perform alternative activities at home show controversial results. In fact, two out of three did not show significant differences between groups [64,67]. On the other hand, in those studies that encourage alternative activities for children within the schools, in addition to alternative strategies for parents, some items that were evaluated with a high risk of bias could influence the results. In fact, the non-possibility to blind participants, personnel, and outcome assessment in most of the articles, or non-allocation concealment in some of them, suggests that researchers should continue working on this research line.

### 4.3. Programs Aimed at a Specific Population

If these interventions are relevant at a time when infants at this vulnerable stage are overexposed to screens, interventions aimed at a specific population are also of interest. This review found two articles that looked for a specific population to which to apply a specific program. These programs are developmental pediatrics clinic setting counseling [61] aimed at children with different neurodevelopmental disorders and the Primary Care Weight Management Program [63] aimed at children with a high or rapidly increasing BMI.

On the one hand, the article developed by Bahadur et al. [61] aimed to investigate the efficacy of counseling in a family-based developmental pediatrics clinical setting to reduce ST in typically developing children and compare it to that of children with neurodevelopmental disorders. To do this, they divided 105 families into two groups: typically developing children (n = 22) and children with a neurodevelopmental disorder (n = 83). Both groups received behavioral counseling for parents about ST, were informed about the effect of ST on the children and were provided with strategies applicable to the children. In the results obtained, there was a statistically significant decrease in ST in both groups after the intervention. The increase in co-viewing percentages and the increase in time spent playing with their children were statistically significant in the neurodevelopmental disorder group. Further, the baseline median screen time before the intervention was 5.0 h a day for all participants (Interquartile range (IQR): 4–9); after the intervention, the median screen time for all participants decreased to 2.0 h a day (IQR:1–3) (*p* < 0.001). Before the intervention, the median percentage spent co-viewing was described as 12.5% of screen time, compared to 40% of screen time after the intervention (*p* = 0.007) for all participants. Thus, it was inferred that the American Academy of Pediatrics media use recommendations are effective in reducing ST in children with typical development or with neurodevelopmental disorders.

On the other hand, Tucker et al. [63] evaluated the impact of a parent-based primary care intervention on health behaviors, including ST, of 2- to 5-year-old children with elevated or rapidly increasing BMI. To do so, he analyzed 165 children by dividing them into two groups: INT group (n = 93) and CON (n = 72). The intervention was based on conversations about healthy behaviors between doctors and families, providing healthy eating behaviors mostly related to food, although they also talked about other types of healthy behaviors, including ST. The results showed an improvement in treatment vs. control (4.6 ± 4.6 vs. 0.1 ± 4.2; *p* < 0.001), and ST (h/day) decreased (−0.9 ± 1.8 vs. 0.3 ± 1.1; *p* < 0.001). In conclusion, families with preschool children who participated in a low-intensity primary care intervention improved children’s ST.

Thus, targeted articles show that interventions tailored to the particular needs of certain groups of children may also be effective in reducing ST. Whether in clinical settings with children with neurodevelopmental disorders or in primary care interventions targeting children with high BMI, these strategies demonstrate that personalized counseling of families, even at low intensity, can lead to positive changes in screen-related habits.

However, the results of the studies included in this section should be specially considered with caution due to the number of included studies and the high risk of bias reported, where both included studies were assessed with high risk of bias in most of the items included in the RoB-2 scale.

### 4.4. Limitations of the Study

The first limitation is that due to the heterogeneity of included studies (contextual factors, countries, ages, parents’ characteristics not detailed in the studies, etc.) and intervention programs. These differences remain in the measurements used in each study. These differences make it difficult to perform a meta-analytical comparison, suggesting future studies are still needed. In addition, due to the limited number of included studies, future research should reinforce the validity and reliability of these intervention programs aimed at reducing ST. In the same way, and although important databases are used, or even databases such as ProQuest Central that include several databases (such as ERIC), future systematic reviews could consider other databases such as SCOPUS or PsycINFO.

## 5. Conclusions

In response to the general objective of the paper, it can be confirmed that programs that require family participation have an impact on the reduction in ST in children under 6 years old. As we have seen, not only programs aimed at parent training but also programs that include activities with children and programs aimed at a specific population have reduced ST in children, almost all of them. On the other hand, in response to the specific objectives of the paper:

• Programs focused on parental training: Items based exclusively on parental training have proven effective as interventions to reduce ST in children under five years of age. Through sessions, educational materials and counseling, it has been possible to modify children’s media habits, both in the short and medium term. Moreover, it has been shown that these interventions are feasible even at very early ages, such as in the youngest children included in the studies. Therefore, parental training from an early stage is a promising strategy to reduce ST in children. Following the included studies, indirect methods to lower children’s ST have been proposed, such as reducing parents’ ST to one hour or fewer per day, using instructional media rather than ads, or reading books every day rather than spending time in front of screens.
• Research based on preparing adults and proposing games with children:
  ○ On the one hand, although the effects of programs focused on the family environment on reducing ST in children are not clear, parental training (through counseling, improvement of parental skills to reduce the child’s ST, suggestions, etc.) contributes to prevent/reduce an increase in ST in children.
  ○ On the other hand, programs that provide training to families to improve the family environment and, at the same time, develop activities with children in schools have been shown to be effective. They have been shown to reduce ST by an average of 25 min, provided that the programs last at least eight months. These results may be useful for professionals working with children in educational settings. However, active parental involvement is essential to achieve lasting and significant reductions in daily screen use. Therefore, it is recommended that these programs be incorporated into primary health care services and at the preschool education stage.
  From these findings, these strategies could be practical to be applied: enhancing parenting techniques while kids engage in self-directed games, advising parents to alter the media environment at home, provide substitutes for ST, and offering advice on parenting techniques while engaging the child in media-free activities of his or her choosing, encouraging parents to try ST through educational lessons or, following classroom instruction and each session, sending materials and activities to promote parent–child interaction at home. All of these recommendations, along with parents’ formation, can help lower children’s ST.
• Programs aimed at a specific population: articles targeting specific populations show that interventions tailored to the specific needs of certain groups of children can be effective in reducing ST. Whether in activities conducted in clinical settings with children with neurodevelopmental disorders or in interventions targeting children with high BMI in primary care, these strategies demonstrate that personalized counseling for families, even of low intensity, can generate positive changes in screen-related habits.

However, these conclusions should be interpreted with caution due to the number of included studies and the large between-study heterogeneity.

## Figures and Tables

**Table 2:** Methodological assessment of the included studies.

Study	Random Sequence Generation	Allocation Concealment	Blinding of Participants and Personnel	Blinding of Outcome Assessment	Incomplete Outcome Data	Selective Reporting	Other Bias
Boonmun et al. [54]	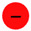	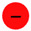	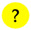	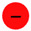	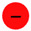	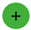	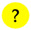
Feng et al. [55]	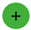	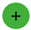	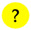	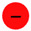	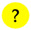	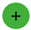	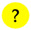
Yilmaz et al. [1]	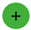	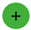	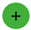	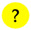	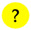	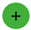	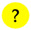
Hinkley et al. [56]	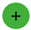	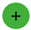	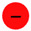	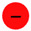	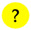	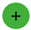	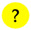
Zimmerman et al. [57]	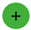	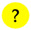	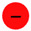	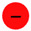	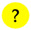	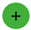	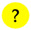
Mendoza et al. [58]	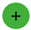	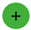	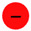	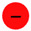	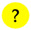	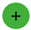	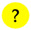
Kaur et al. [59]	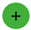	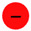	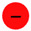	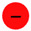	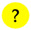	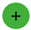	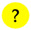
Raj et al. [60]	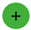	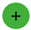	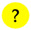	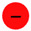	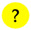	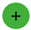	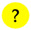
Bahadur et al. [61]	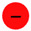	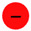	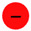	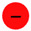	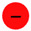	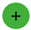	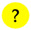
Poonia et al. [62]	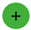	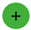	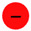	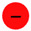	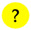	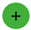	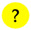
Tucker et al. [63]	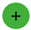	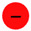	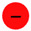	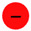	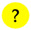	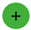	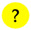
Tuominen et al. [64]	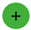	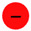	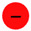	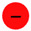	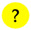	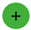	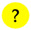
Romo and Abril-Ulloa [65]	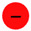	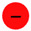	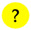	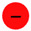	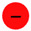	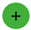	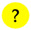
Dennison et al. [66]	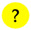	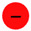	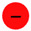	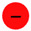	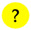	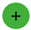	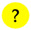
Birken et al. [67]	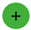	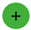	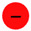	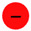	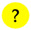	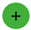	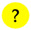

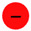
 High risk; 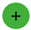 low risk; 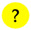 some concerns.

## Data Availability

The data presented in this study are available on request from the corresponding author due to (specify the reason for the restriction, e.g., privacy, legal or ethical reasons).

## Supplementary Materials

The following supporting information can be downloaded at: https://www.mdpi.com/article/10.3390/children13040446/s1. S1: Characteristics of included studies; S2: Characteristics of intervention programs.

## References

[1] Yilmaz G., Demirli Caylan N., Karacan C.D. (2015). An Intervention to Preschool Children for Reducing Screen Time: A Randomized Controlled Trial. Child.

[2] Byrne R., Terranova C.O., Trost S.G. (2021). Measurement of Screen Time among Young Children Aged 0–6 Years: A Systematic Review. Obes. Rev..

[3] Paz-Cantos S.D., González-Marrón A., Lidón-Moyano C., Cerrato-Lara M., Díez-Izquierdo A., Martínez-Sánchez J.M. (2024). Smartphone and Tablet Use Pattern in Children up to 5 Years Old in Spain: A Cross-Sectional Study. Rev. Lat.-Am. Enferm..

[4] Lumeng J.C., Miller A.L., Horodynski M.A., Brophy-Herb H.E., Contreras D., Lee H., Sturza J., Kaciroti N., Peterson K.E. (2017). Improving Self-Regulation for Obesity Prevention in Head Start: A Randomized Controlled Trial. Pediatrics.

[5] Pedrouzo S.B., Peskins V., Garbocci A.M., Sastre S.G., Wasserman J. (2020). Screen Use among Young Children and Parental Concern. Arch. Argent. Pediat..

[6] Chaput J.-P., Willumsen J., Bull F., Chou R., Ekelund U., Firth J., Jago R., Ortega F.B., Katzmarzyk P.T. (2020). 2020 WHO Guidelines on Physical Activity and Sedentary Behaviour for Children and Adolescents Aged 5–17 Years: Summary of the Evidence. Int. J. Behav. Nutr. Phys. Act..

[7] Scarborough P., Bhatnagar P., Wickramasinghe K.K., Allender S., Foster C., Rayner M. (2011). The Economic Burden of Ill Health Due to Diet, Physical Inactivity, Smoking, Alcohol and Obesity in the UK: An Update to 2006-07 NHS Costs. J. Public Health.

[8] Piercy K.L., Troiano R.P. (2018). Physical Activity Guidelines for Americans From the US Department of Health and Human Services. Circ. Cardiovasc. Qual. Outcomes.

[9] Eckermann S., Willan A.R. (2022). Active Lives South Australia Health Economic Analysis: An Evidence Base for the Potential of Health Promotion Strategies Supporting Physical Activity Guidelines to Reduce Public Health Costs While Improving Wellbeing. J. Public Health.

[10] Tremblay M.S., Leblanc A.G., Carson V., Choquette L., Connor Gorber S., Dillman C., Duggan M., Gordon M.J., Hicks A., Janssen I. (2012). Canadian Physical Activity Guidelines for the Early Years (Aged 0–4 Years). Appl. Physiol. Nutr. Metab..

[11] Arufe-Giráldez V., Sanmiguel-Rodríguez A., Zagalaz-Sánchez M.L., Cachón-Zagalaz J., González-Valero G. (2022). Sleep, Physical Activity and Screens in 0–4 Years Spanish Children during the COVID-19 Pandemic: Were the WHO Recommendations Met?. J. Hum. Sport Exerc..

[12] Okely A.D., Ghersi D., Hesketh K.D., Santos R., Loughran S.P., Cliff D.P., Shilton T., Grant D., Jones R.A., Stanley R.M. (2017). A Collaborative Approach to Adopting/Adapting Guidelines—The Australian 24-Hour Movement Guidelines for the Early Years (Birth to 5 Years): An Integration of Physical Activity, Sedentary Behavior, and Sleep. BMC Public Health.

[13] Sommer I., Nußbaumer-Streit B., Gartlehner G. (2021). WHO Guideline: Physical Activity, Sedentary Behavior and Sleep for Children under 5 Years of Age. Gesundheitswesen.

[14] Tremblay M.S. (2020). Introducing 24-Hour Movement Guidelines for the Early Years: A New Paradigm Gaining Momentum. J. Phys. Act. Health.

[15] Aadland E., Tjomsland H.E., Johannessen K., Nilsen A.K.O., Resaland G.K., Glosvik Ø., Lykkebø O., Stokke R., Andersen L.B., Anderssen S.A. (2020). Active Learning Norwegian Preschool(Er)s (ACTNOW)—Design of a Cluster Randomized Controlled Trial of Staff Professional Development to Promote Physical Activity, Motor Skills, and Cognition in Preschoolers. Front. Psychol..

[16] Rico-González M., Goth U.S., Martín-Moya R., Ardigò L.P. (2025). The Relationship with Meeting Physical Activity Guidelines in Preschool-Aged Children: A Systematic Review. Pediatr. Rep..

[17] Downing K.L., Hnatiuk J.A., Hinkley T., Salmon J., Hesketh K.D. (2018). Interventions to Reduce Sedentary Behaviour in 0–5-Year-Olds: A Systematic Review and Meta-Analysis of Randomised Controlled Trials. Br. J. Sports Med..

[18] LeBlanc A.G., Spence J.C., Carson V., Connor Gorber S., Dillman C., Janssen I., Kho M.E., Stearns J.A., Timmons B.W., Tremblay M.S. (2012). Systematic Review of Sedentary Behaviour and Health Indicators in the Early Years (Aged 0–4 Years). Appl. Physiol. Nutr. Metab..

[19] Janssen X., Martin A., Hughes A.R., Hill C.M., Kotronoulas G., Hesketh K.R. (2020). Associations of Screen Time, Sedentary Time and Physical Activity with Sleep in under 5s: A Systematic Review and Meta-Analysis. Sleep Med. Rev..

[20] Suchert V., Hanewinkel R., Isensee B. (2015). Sedentary Behavior and Indicators of Mental Health in School-Aged Children and Adolescents: A Systematic Review. Prev. Med..

[21] Wu X., Tao S., Rutayisire E., Chen Y., Huang K., Tao F. (2017). The Relationship between Screen Time, Nighttime Sleep Duration, and Behavioural Problems in Preschool Children in China. Eur. Child Adolesc. Psychiatry.

[22] Wang H., Zhao J., Yu Z., Pan H., Wu S., Zhu Q., Dong Y., Liu H., Zhang Y., Jiang F. (2024). Types of On-Screen Content and Mental Health in Kindergarten Children. JAMA Pediatr..

[23] Muppalla S.K., Vuppalapati S., Reddy Pulliahgaru A., Sreenivasulu H. (2023). Effects of Excessive Screen Time on Child Development: An Updated Review and Strategies for Management. Cureus.

[24] Mougharbel F., Goldfield G.S. (2020). Psychological Correlates of Sedentary Screen Time Behaviour Among Children and Adolescents: A Narrative Review. Curr. Obes. Rep..

[25] Stiglic N., Viner R.M. (2019). Effects of Screentime on the Health and Well-Being of Children and Adolescents: A Systematic Review of Reviews. BMJ Open.

[26] Gath M., Horwood L.J., Gillon G., McNeill B., Woodward L.J. (2025). Longitudinal Associations between Screen Time and Children’s Language, Early Educational Skills, and Peer Social Functioning. Dev. Psychol..

[27] Robin A., Padmanabhan V., Swaminathan K., Kc V., K V., Haridoss S. (2025). Association Between Screen Time, Dietary Patterns, and Oral Health Among Children: A Cross-Sectional Study. Cureus.

[28] Shqair A.Q., Pauli L.A., Costa V.P.P., Cenci M., Goettems M.L. (2019). Screen Time, Dietary Patterns and Intake of Potentially Cariogenic Food in Children: A Systematic Review. J. Dent..

[29] Wu Y., Xi X., Zhang C., Jiang J., Ye S. (2025). Effect of Intervention on Screen Time in Preschoolers: A Systematic Review and Meta-Analysis of Randomized Controlled Trials. BMC Public Health.

[30] Rico-González M., Holsbrekken E., Martín-Moya R., Ardigò L.P. (2025). Interventions for Reducing Screen Time of Preschoolers: A Systematic Review of Randomized Controlled Trials. J. Prim. Care Community Health.

[31] Streegan C.J.B., Lugue J.P.A., Morato-Espino P.G. (2022). Effects of Screen Time on the Development of Children under 9 Years Old: A Systematic Review. J. Pediatr. Neonatal Individ. Med..

[32] Raj D., Mohd Zulkefli N.A., Minhat H.S., Ahmad N. (2022). Parental Intervention Strategies to Reduce Screen Time Among Preschool-Aged Children: A Systematic Review. MJMHS.

[33] Modecki K.L., Goldberg R.E., Wisniewski P., Orben A. (2022). What Is Digital Parenting? A Systematic Review of Past Measurement and Blueprint for the Future. Perspect. Psychol. Sci..

[34] Mallawaarachchi S. (2024). Early Childhood Screen Use Contexts and Cognitive and Psychosocial Outcomes: A Systematic Review and Meta-Analysis. JAMA Pediatr..

[35] Flaibam Giovanelli J., Soares Da Silva L., Abadio De Oliveira W., Scatena A., Ferreira Semolini F., Monezi Andrade A.L. (2025). Parental Mediation in the Use of Screens by Children and Adolescents: A Systematic Literature Review. CienciasPsi.

[36] Sevilla-Fernández D., Díaz-López A., Caba-Machado V., Machimbarrena J.M., Ortega-Barón J., González-Cabrera J. (2025). Parental Mediation and the Use of Social Networks: A Systematic Review. PLoS ONE.

[37] Zgambo M., Anyango E., Arabiat D.H., Ngune I., Mörelius E., Zhang M., Whitehead L.C. (2025). Effect of Digital Safety Interventions on Parental Practices in Safeguarding Children’s Digital Activities: Systematic Review and Meta-Analysis. JMIR Pediatr. Parent..

[38] Torres P.E., Ulrich P.I.N., Cucuiat V., Cukurova M., Fercovic De La Presa M.C., Luckin R., Carr A., Dylan T., Durrant A., Vines J. (2021). A Systematic Review of Physical–Digital Play Technology and Developmentally Relevant Child Behaviour. Int. J. Child-Comput. Interact..

[39] Staiano A.E., Webster E.K., Allen A.T., Jarrell A.R., Martin C.K. (2018). Screen-Time Policies and Practices in Early Care and Education Centers in Relationship to Child Physical Activity. Child. Obes..

[40] Zhang Z., Adamo K.B., Ogden N., Goldfield G.S., Okely A.D., Kuzik N., Crozier M., Hunter S., Predy M., Carson V. (2022). Associations between Screen Time and Cognitive Development in Preschoolers. Paediatr. Child Health.

[41] Bracho-Sanchez E. Screen Time for Kids Under 2 More than Doubles, Study Finds. CNN Health 2019. https://edition.cnn.com/2019/02/18/health/kids-screen-time-tv-study/index.html.

[42] Suárez Palacio P.A., Vélez Múnera M. (2018). El papel de la familia en el desarrollo social del niño: Una mirada desde la afectividad, la comunicación familiar y estilos de educación parental. Psicoespacios.

[43] Varela Arévalo M.T., Tenorio Banguero Á.X., Duarte Alarcón C. (2018). Prácticas parentales para promover hábitos saludables de alimentación en la primera infancia en Cali, Colombia. Rev. Esp. Nutr. Hum. Diet..

[44] Ding X., Ji Y., Dong Y., Li Z., Zhang Y. (2024). The Impact of Family Factors and Communication on Recreational Sedentary Screen Time among Primary School-Aged Children: A Cross-Sectional Study. BMC Public Health.

[45] Dava T.Y., Cecibel G., Cedeño M.J., Barcia M.F. (2023). Orientación familiar en el desarrollo de autonomía en niños de educación inicial. Pol. Con..

[46] Hmidan A., Seguin D., Duerden E.G. (2023). Media Screen Time Use and Mental Health in School Aged Children during the Pandemic. BMC Psychol..

[47] Veldman S.L.C., Altenburg T.M., Chinapaw M.J.M., Gubbels J.S. (2023). Correlates of Screen Time in the Early Years (0–5 Years): A Systematic Review. Prev. Med. Rep..

[48] Csima M., Podráczky J., Keresztes V., Soós E., Fináncz J. (2024). The Role of Parental Health Literacy in Establishing Health-Promoting Habits in Early Childhood. Children.

[49] Mekhail K.T., Blom L., Rydström L.-L. (2024). Young Children’s Screen Habits and First-Time Parents’ Reflections on Screen Use in Socioeconomically Disadvantaged Swedish Settings: A Mixed Methods Study. BMC Public Health.

[50] Trubey R.J., Moore S.C., Chestnutt I.G. (2015). Children’s Toothbrushing Frequency: The Influence of Parents’ Rationale for Brushing, Habits and Family Routines. Caries Res..

[51] Rico-González M., Pino-Ortega J., Clemente F.M., Los Arcos A. (2022). Guidelines for Performing Systematic Reviews in Sports Science. Biol. Sport..

[52] Page M.J., McKenzie J.E., Bossuyt P.M., Boutron I., Hoffmann T.C., Mulrow C.D., Shamseer L., Tetzlaff J.M., Akl E.A., Brennan S.E. (2021). The PRISMA 2020 Statement: An Updated Guideline for Reporting Systematic Reviews. BMJ.

[53] Sterne J.A.C., Savović J., Page M.J., Elbers R.G., Blencowe N.S., Boutron I., Cates C.J., Cheng H.-Y., Corbett M.S., Eldridge S.M. (2019). RoB 2: A Revised Tool for Assessing Risk of Bias in Randomised Trials. BMJ.

[54] Boonmun W., Phuphaibul R., Hongsanguansri S., Nookong A., Chansatitporn N. (2023). A Program for Parents’ Screen Time Reduction for Preschool Children: A Quasi-Experimental Study. PRIJNR.

[55] Feng J., Huang W.Y., Sit C.H.-P., Reilly J.J., Khan A. (2024). Effectiveness of a Parent-Focused Intervention Targeting 24-Hour Movement Behaviours in Preschool-Aged Children: A Randomised Controlled Trial. Int. J. Behav. Nutr. Phys. Act..

[56] Hinkley T., Cliff D.P., Okely A.D. (2015). Reducing Electronic Media Use in 2–3 Year-Old Children: Feasibility and Efficacy of the Family@play Pilot Randomised Controlled Trial. BMC Public Health.

[57] Zimmerman F.J., Ortiz S.E., Christakis D.A., Elkun D. (2012). The Value of Social-Cognitive Theory to Reducing Preschool TV Viewing: A Pilot Randomized Trial. Prev. Med..

[58] Mendoza J.A., Baranowski T., Jaramillo S., Fesinmeyer M.D., Haaland W., Thompson D., Nicklas T.A. (2016). Fit 5 Kids TV Reduction Program for Latino Preschoolers. Am. J. Prev. Med..

[59] Kaur N., Gupta M., Chakrapani V., Khan F., Malhi P., Kiran T., Grover S. (2024). Effectiveness of a Program to Lower Unwanted Media Screens among 2–5-Year-Old Children: A Randomized Controlled Trial. Front. Public Health.

[60] Raj D., Ahmad N., Mohd Zulkefli N.A., Lim P.Y. (2023). Stop and Play Digital Health Education Intervention for Reducing Excessive Screen Time Among Preschoolers From Low Socioeconomic Families: Cluster Randomized Controlled Trial. J. Med. Internet Res..

[61] Bahadur E.İ., Akkuş P.Z., Yoldaş T.Ç., Özmert E.N. (2021). How Effective Is Family Counselling on Screen Exposure of Pre-School Children?. Turk. J. Pediatr..

[62] Poonia Y., Khalil S., Meena P., Shah D., Gupta P. (2024). Parental Education for Limiting Screen Time in Early Childhood: A Randomized Controlled Trial. Indian Pediatr..

[63] Tucker J.M., DeFrang R., Orth J., Wakefield S., Howard K. (2019). Evaluation of a Primary Care Weight Management Program in Children Aged 2–5 Years: Changes in Feeding Practices, Health Behaviors, and Body Mass Index. Nutrients.

[64] Tuominen P.P.A., Husu P., Raitanen J., Kujala U.M., Luoto R.M. (2017). The Effect of a Movement-to-Music Video Program on the Objectively Measured Sedentary Time and Physical Activity of Preschool-Aged Children and Their Mothers: A Randomized Controlled Trial. PLoS ONE.

[65] Romo M.L., Abril-Ulloa V. (2018). Improving Nutrition Habits and Reducing Sedentary Time Among Preschool-Aged Children in Cuenca, Ecuador: A Trial of a School-Based Intervention. Prev. Chronic Dis..

[66] Dennison B.A., Russo T.J., Burdick P.A., Jenkins P.L. (2004). An Intervention to Reduce Television Viewing by Preschool Children. Arch. Pediatr. Adolesc. Med..

[67] Birken C.S., Maguire J., Mekky M., Manlhiot C., Beck C.E., DeGroot J., Jacobson S., Peer M., Taylor C., McCrindle B.W. (2012). Office-Based Randomized Controlled Trial to Reduce Screen Time in Preschool Children. Pediatrics.

[68] Khoo C.-S., Ramachandram S. (2022). The Effect of Parent Training Programmes on Screen Time and Social Function in Children with Autism Spectrum Disorder. MJMS.

[69] Li C., Cheng G., Sha T., Cheng W., Yan Y. (2020). The Relationships between Screen Use and Health Indicators among Infants, Toddlers, and Preschoolers: A Meta-Analysis and Systematic Review. Int. J. Environ. Res. Public Health.

[70] Rico-González M., González-Devesa D., Gómez-Carmona C.D., Moreno-Villanueva A. (2025). Exercise as Modulator of Brain-Derived Neurotrophic Factor (BDNF) in Children: A Systematic Review of Randomized Controlled Trials. Life.

