# Supplementary material for: The Effects of Family-Based Programs on Preschool Children’s Screen Time: A Systematic Review
Source: Children (Basel). 2026 Mar 25;13(4):446. doi: 10.3390/children13040446 (PMC13114308; doi:10.3390/children13040446)
Supplement: Supplementary file 1 [file children-13-00446-s001.zip › Supplementary material S1.pdf]

Table. Characteristics of included studies

Reference	Aim	Sample	Intervention: Group differences	Intervention: Duration	Results: Test instrument	Results: Results	Highlights
**Parents` formation**							
Boonmun et al. [54]	To examine the effects of the newly developed PSTRPP, a training program for parents' planned behaviours and the ST reduction of their children	Dyads of parents and preschool children aged 2-5 years, where the child's ST was more than one hour per day	INT group<br>n: 35<br>Program: PSTRPP<br><br>CON group<br>n: 32<br>Program: -	Three sessions, three hours long, over two weeks.	- The Parents' Attitudes regarding Children's ST Questionnaire<br>- The Parents' Subjective Norms regarding Children's ST Reduction Questionnaire<br>- The Parents' Perceived Behavioural Control concerning Children's ST Reduction Questionnaire<br>- The Parents' Intentions concerning Children's ST Reduction Questionnaire<br>- The Parents' Behaviours concerning Children's ST Reduction Questionnaire	The findings revealed that the children's ST in the experimental group decreased significantly more at one week and two months after the completion of the intervention than that of the control group.<br><br>The findings of this study showed children's ST in the EXP significantly differed from those in the CON ($p < 0.05$).<br>Children in the EXP exhibited lower ST than those in the CON at post test 1 and 2. Additionally, children's ST in the practical decreased from 127.14 minutes/day to 70.64 minutes/day at post test 1 and 77.14 minutes/ day at post test 2.<br><br>Regression coefficient showed:<br>Post test 1: -49.36 ± 9.55 (95%CI: from -68.07 to -30.64; p<.001)<br>Post test 1: -37.31 ± 11.43 (95%CI: from -59.31 to -14.91; p.001)	Nurses and teachers can use this program among parents to perform appropriate ST behaviours to promote development and health outcomes among children.
Feng et al. [55]	To assess the effectiveness of an intervention aimed at improving all three 24-hour movement behaviours among preschoolers	147 parent-child pair:<br>- 111 parent-child pairs from eight kindergartens<br>- 36 individually recruited pairs.	INT group<br>n: 49<br>Program: Integrated approach. Parent-focused intervention.<br><br>Received education material about: individual reports on their children's current behaviours, gaps and recommendations to improve behaviours, habit development.	24 weeks	- Accelerometers<br>- Biweekly interactive questionnaires using Google Forms	Preschoolers in both intervention groups had a decreased ST at post-intervention and follow-up.<br><br>In the integrated focus group: A reduction of 33 minutes per day in ST was observed compared to the CON, with a small to moderate effect size ($d = 0.42$) post-intervention and a small effect size ($d = 0.27$) at follow-up.<br><br>In the dyadic focus group: A reduction of 31 minutes per day in ST was observed	Both intervention groups showed a decrease in ST at post-intervention, but there were no significant changes in other behaviours. The favourable changes observed at follow-up demonstrated the effectiveness of both intervention approaches on alleviating the decline in the composition of reducing ST and revealed the possible effectiveness of the integrated approach in promoting

Reference	Aim	Sample	Intervention		Results		Highlights
			Group differences	Duration	Test instrument	Results	
			A workshops was included<br>INT group<br>n: 47<br>Program: dyadic approach. Parent-focused intervention<br><br>The same intervention contents as the integrated approach, with the exception that the intervention materials were limited to PA and SB (including sedentary ST).<br>CON group<br>n: 51<br>Program: without intervention			compared to the CON, with a moderate to large effect size (d = 0.66) post-intervention and a large effect size (d = 0.75) at follow-up.	overall movement behaviours among preschoolers.<br><br>Both programs were effective in reducing ST, with the dyadic approach showing the largest effect size compared to the integrated approach.
Yilmaz et al. [1]	To determine if a simple intervention aimed at preschool-aged children, applied at the health maintenance visits, in the primary care setting, would be effective in reducing ST	363 families:<br>2-6 year-old children and their parents	INT group<br>n: 187 families<br>program: intervention for reducing ST<br>CON group<br>n: 176 families<br>Program: the families in the control group were not aware of counselling interventions	2 years	- The test was made at baseline at the 2nd, 6th and 9th months. The parents completed a shorter questionnaire. In this questionnaire they reported their children's<br>- TV/video watching and computer/video game playing. The parents kept a record about the length of ST of their children for 1 week	Parents in the INT reported less ST than those in the CON	This study shows that a preschool-based intervention can lead to reductions in young children's TV/video viewing
Hinkley et al. [56]	To test the feasibility and potential efficacy of a family-based program to decrease ED use in 2–3-year-old children	Parents of 2–3 year-old children	INT group<br>n: 12<br>Program: Family@play<br>CON group<br>n: 10	2 years	- Parents completed a time-use diary on each of four days and a survey<br>-Accelerometer.	Process evaluation results were high, showing the acceptability of the program. Compared with children in the CON, there were greater decreases in total ED use among children in the intervention group (adjusted difference [95 % CI] = −31.2 mins/day [−71.0–8.6] Cohen's d = 0.70). Differences for other outcomes were in the hypothesised direction and ranged from small to moderate to large	Family@play was shown to be a feasible and acceptable intervention to deliver to families of 2–3 year old children. Potential efficacy is evident from moderate to large effect sizes. A larger trial is warranted to test the efficacy of the program.

Reference	Aim	Sample	Intervention		Results		Highlights
			Group differences	Duration	Test instrument	Results	
			Program: attended the same sessions following the collection of all follow-up data			for individual ED (e.g. TV viewing, DVD/video viewing).	
Zimmerman et al. [57]	To assess the effectiveness of a program to (a) reduce the total amount of TV viewing to which preschool children are exposed; (b) shift the balance of exposure away from commercial TV toward educational content.	67 families. Children from 2 to 5 years	INT group<br>n: 60<br>Program: Parental Education for Limiting ST in Early Childhood<br>CON group<br>n: 60<br>Program: received routine counselling regarding nutrition, immunization, and general safety measures.	2008-2011	Questionnaire about the amount of total TV viewing and the amount of commercial TV/DVD/video viewing of the child	Compared to those in the CON, families randomized to the INT experienced a significant reduction by 37 minutes/day in total viewing time (95% CI: 5.6–68.7), including a marginally significant reduction by 29 minutes/day in viewing of commercial content (95% CI: −4.6–63)	Thus, substitution of educational media as an alternative strategy may be an effective behavioural intervention to achieve public health objectives in this population
Poonia et al. [62]	To assess the impact of focused parental education on limiting ST in early childhood.	120 healthy children (9 -10 months of age)	INT group<br>n: 60<br>Program: educational group<br>CON group<br>n: 60<br>Program: no name	6 months	Questionnaire about:<br>-Household ownership of TV, handheld devices like smartphones, tablets, laptops, and personal computers was also ascertained.<br>-Frequency of screen viewing practices of their child (days/week).<br>-Child's age at first exposure to screen devices was ascertained and the primary caregiver's screen-time (frequency and duration in a week) were also documented.<br>- Ask about the primary caregiver about perception of their own and their child's screen viewing habits.<br>-involvement in activities like watching/using screen device during meal time, for entertainment and academic activities.	Children in the INT had ST > 1 hour/day as compared to 53% (32/60) ($p < 0.001$) in the CON. Median (IQR) for total screen duration in the Educational group was 35 (30,49) minutes/day compared to 75 (50,90) minutes/day in the CON ($p < 0.001$).	Parental education starting in infancy is a promising intervention to reduce screen exposure in children

Reference	Aim	Sample	Intervention		Results		Highlights
			Group differences	Duration	Test instrument	Results	
**Parents formation and Alternative Games with Children**							
Mendoza et al. [58]	To evaluate the F5K TV reduction program's impact on Latino preschooler's TV viewing.	160 children aged 3–5 years and their parents	INT group<br>n: 90<br>Program: F5K TV reduction program's<br><br>CON group<br>n: 70<br>Program: received the usual Head Start general curriculum taught by Head Start teachers, which did not specifically include lessons or materials on limiting TV viewing.	2 years	- Outcome variables and covariates were obtained at the Head Start centres or participant homes (TV diaries) and pertained to the individual-level measurements.<br>-Accelerometers	Per the adjusted repeated measures linear mixed effects model for TV viewing (minutes/day), intervention children decreased from 76.2 (9.9) at Time 1 to 52.1 (10.0) at Time 2, whereas control children remained about the same from 84.2 (10.5) at Time 1 to 85.4 (10.5) at Time 2. The relative difference from Time 1 to Time 2 was –25.3 (95% CI= –45.2, –5.4) minutes for intervention vs control children (N=160, p=0.01). In a similar adjusted model, there was a relative decrease in sedentary time (minutes/day) from Time 1 to Time 2 favoring the intervention children (–9.5, 95% CI= –23.0, 4.1), although not significant at p<0.05	F5K reduced Latino preschoolers' TV viewing by >25 minutes daily. These findings have implications for prevention of obesity, related disorders, and health equity
Kaur et al. [59]	To develop and assess the effectiveness of the PLUMS to lower unwanted media ST	340 families and their children (2 - 5 (±3 months) years old)	INT group<br>n: 170<br>Program: PLUMS<br><br>CON group<br>n: 170<br>Program: received routine healthcare services.	8 months	Digital-screen exposure questionnaire	The mean difference in ST on a typical day [27.7 min, 95% CI 5.1, 50.3] at the post-intervention assessment significantly (p < 0.05) decreased in the intervention (102.6 ± 98.5 min) arm as compared with the control (130.3 ± 112.8 min) arm.<br>A significant reduction in ST (β = −35.81 min, CI -70.6, −1.04) from baseline (β = 123.1 min) to follow-up phase (β = 116 min) was observed in GEE analysis.	The PLUMS intervention significantly reduced the children's mean ST on a typical day. These results might guide the policymakers to include strategies in the national child health programs in the Southeast Asia Region to reduce unwanted ST.
Raj et al. [60]	To develop, implement, and evaluate the effectiveness of Stop and Play, a digital parental health education intervention to reduce excessive ST among preschoolers from low	360 mother-child dyads aged 3 to 4 years<br>And 16 clusters	INT group<br>n: 180 mother-child dyads<br>Program: Stop and Play<br><br>CON group<br>n: 180 mother-child dyads<br>Program: recruitment and progress of all participants	March 2021 and December 2021.	- SCREENS questionnaire<br>- Mother's Perception About the Influence of ST on a Child's Well-being<br>- Mother's Self-efficacy: Mother's self-efficacy to reduce ST<br>- Mother's ST	At 3 months after the intervention, the INT showed significantly reduced child's ST compared with the control group (β=−202.29, 95% CI −224.48 to −180.10; P<.001). Mother's knowledge significantly increased (β=6.88, 95% CI 6.11-7.65; P<.001), whereas perception about the influence of ST on the child's well-being reduced (β =−.86, 95% CI	The Stop and Play intervention was effective in reducing ST among preschool children from low socioeconomic families, while improving the associated parental factors. Therefore, integration into primary health care and preschool education programs is

Reference	Aim	Sample	Intervention		Results		Highlights
			Group differences	Duration	Test instrument	Results	
	socioeconomic families in Malaysia		throughout the study period.		- Physical Household Environment: to report if digital devices were present in the child's bedroom	−0.98 to −0.73; P<.001). There was also an increase in the mother's self-efficacy to reduce ST (β =1.59, 95% CI 1.48-1.70; P<.001) and increase PA (β =.07,95% CI 0.06-0.09; P<.001), along with reduction in mother's ST (β =−70.43, 95% CI −91.51 to −49.35; P<.001)	recommended. Mediation analysis is suggested to investigate the extent to which secondary outcomes are attributable to the child's ST, and long follow-up could evaluate the sustainability of this digital intervention
Tuominen et al. [64]	To determine whether a movement-to-music video program could reduce SB and increase PA in mother-child pairs in the home environment.	203 mother-child pairs (child age 5±7 years)	INT group<br>n: 102<br>Program: "Movement-to-music video program"<br><br>CON group<br>n: 101<br>Program: -	2 years	- Questionnaires	No statistically significant differences between groups were found in primary or secondary outcomes. Among the children in the CON, ST increased from 89 (SD 37) to 99 (SD 41) min/d. Among mothers and children in the intervention group, no statistical differences were found.<br>In supplementary analysis, the children who stayed at home instead of attending day care/ preschool had on average 25 (95%, CI 19±30) min/d more sedentary time.	The movement-to-music video program did not change objectively measured SB. However, mothers and children seem to be more sedentary at home than at work and preschool or day care, and therefore, interventions to decrease SB should be targeted especially at the home environment. In addition, for those mothers and young children who have difficulties in exercising outside the home, the movement-to-music video program might represent a way to be physically active.
Romo & Abril-Ulloa [65]	The objective of this study was to implement and evaluate this intervention to improve nutrition and PA habits.	307 families and their children /3 – 4 years old)	INT group (pilot intervention)<br>n: 155<br>Program: behavioral interventions to improve nutrition and physical activity habits (3 months at school)<br><br>INT group (enhanced intervention)<br>n: 152<br>Program: behavioral interventions to improve nutrition and physical activity habits (7 months at both school and home)	2 years	- Questionnaires to the parent or guardian about children's at-home nutrition habits, sedentary time, and anthropometry.	Additional beneficial effects of the Enhanced Intervention not observed with the Pilot Intervention were a reduction in excessive weekend ST (−7.6%, P = .03)	Our preschool-based intervention appeared to be successful in promoting healthy lifestyle habits, especially when combined with a household component

Reference	Aim	Sample	Intervention		Results		Highlights
			**Group differences**	**Duration**	**Test instrument**	**Results**	
Dennison et al. [66]	To develop and evaluate an intervention to reduce TV viewing by preschool children.	Children aged 2.5 through 5.5 years who attended a participating preschool or day care centre	INT group<br>n: 93<br>Program: intervention to reduce children`s TV viewing<br><br>CON group<br>n: 83<br>Program: received a safety and injury prevention program.	Once a week to provide a 1-hour session for a total of 39 weeks.	- Questionnaires during week and weekend about the current and past habits about time viewing screens (TV or videos, playing video or computer games, or surfing the Internet).	Before the intervention, the intervention and control groups viewed 11.9 and 14.0 h/wk of TV/ videos, respectively. Afterward, children in the intervention group decreased their TV/video viewing 3.1 h/wk, whereas children in the control group increased their viewing by 1.6 h/wk, for an adjusted difference between the groups of −4.7 h/wk (95% CI, −8.4 to −1.0 h/wk; P=.02). The percentage of children watching TV/videos more than 2 h/d also decreased significantly from 33% to 18% among the intervention group, compared with an increase of 41% to 47% among the CON, for a difference of −21.5% (95%CI, −42.5% to −0.5%; P=.046)	This study is the first to show that a preschool- based intervention can lead to reductions in young children's TV/video viewing. Further research is needed to determine the long-term effects associated with reductions in young children's TV viewing
Birken et al. [67]	determine if an intervention for preschool-aged children in primary care is effective in reducing ST and meals in front of the TV	160 families of three-year-old children and their parents	INT group<br>n: 81<br>Program: intervention to reduce children's television viewing<br><br>INT group<br>n: 79<br>Program: no detailed	3 months	- Questionnaires	In the intention-to-treat analysis at 1 year, there were no significant differences in mean total weekday minutes of ST (60, interquartile range [IQR]: 35–120 vs 65, IQR: 35–120; p = .68) or mean total weekend day minutes of ST (80, IQR: 45–130 vs 90, IQR: 60–120; p = 0.33) between the intervention and control group. Adjusting for baseline BMI, there was a reduction in the number of weekday meals in front of the TV (1.6 6 1.0 vs 1.9 6 1.2; p = .03) but no differences in BMI or number of TVs in the bedroom	This pragmatic trial was not effective in reducing ST but was effective in reducing meals in front of the screen. Short interventions focused solely on reducing ST implemented in the primary care practice setting may not be effective in this age group
**Specific population**							
Bahadur et al. [61]	To investigate the effectiveness of family-based, developmental pediatrics clinic setting counseling in reducing ST in	105 children (aged 24-62 months) who were exposed to screen viewing for at least 2 hours/day were included.	INT group 1<br>n: 22 children with a typical development<br>Program: developmental pediatrics clinic setting counseling	3 years	ST, background TV exposure, co-viewing, promotion development activities, feeding difficulties were collected by a questionnaire specifically designed and asked for the study by the researchers.	There was a statistically significant decrease in ST in both groups after the intervention.<br>The increase in percentages of co-viewing, as well as the increase in the time spent playing with their children,	The study demonstrated that three paediatric office-setting counselling sessions including media use recommendations of the American Academy of Paediatrics are effective to decrease ST for

Reference	Aim	Sample	Intervention		Results		Highlights
			Group differences	Duration	Test instrument	Results	
	typically developing children and to compare them with neurodevelopmental disorders.		INT group 2<br>n: 2 private paediatric office<br>Program: Primary Care Weight Management			were statistically significant in the neurodevelopmental disorder group.<br><br>In fact, the baseline median screen time before the intervention was 5.0 hours a day for all participants (Interquartile range (IQR): 4-9), after the intervention the median screen time for all participants decreased to 2.0 hours a day (IQR:1-3) (p<0.001). Before the intervention, the median percentage spent co-viewing was described as 12.5% of screen time, compared to 40% of screen time after the intervention (p=0.007) for all participants.	children who are either typically developing or with a neurodevelopmental disorder.
Tucker et al. [63]	Assessed the impact of a parent-based, primary care intervention on the health behaviors (ST), of 2–5 year olds with elevated or rapidly-increasing BMI.	(n=165) children 2–5 years	INT group<br>n: 83 with a neurodevelopmental disorder<br>Program:<br><br>CON group<br>n: 2 private paediatric office<br>Program: received their usual medical care	6 month	- Child/Family Behaviors lifestyle questionnaire: ST (including watching TV, playing video games, and using cell phones, tablets, and computers)<br>- (FNPA) screening tool: Included, family eating habits (TV during meals and fast food consumption) and ST (TV/video game quantity and limit setting), healthy environment (TV in the bedroom and PA opportunities)	FNPA scores improved in treatment vs. control (4.6 ± 4.6 vs. 0.1 ± 4.2; p < 0.001), and ST (h/day) decreased (-0.9 ± 1.8 vs. 0.3 ± 1.1; p < 0.001).	Families with preschool children participating in a low-intensity, primary care intervention improved child ST. Future research should assess the sustainability of these family lifestyle improvements, and evaluate their future impact on the health and development of the children.

Note: BMI: Body mass index; CON: control; ED: electronic device; F5K: Fit 5 Kids; FNPA: Family Nutrition and Physical Activity; GEE: Generalized estimating equation; INT: intervention; PA: physical activity; PLUMS: Program to lower unwanted media screen; PSTRPP: Parents' Screen Time Reduction for Preschool Children Program; SB: Sedentary behaviour; ST: screen time; TV: television
