# Supplementary material for: The Effects of Family-Based Programs on Preschool Children’s Screen Time: A Systematic Review
Source: Children (Basel). 2026 Mar 25;13(4):446. doi: 10.3390/children13040446 (PMC13114308; doi:10.3390/children13040446)
Supplement: Supplementary file 1 [file children-13-00446-s001.zip › Supplementary material S2.pdf]

Supplementary material S2. Characteristics of intervention programs

PROGRAM	INTERVENTION MAIN IDEAS		
PLUMS	GENERAL INFORMATION	MODULES	
		FOR PARENTS	FOR CHILDREN
	The strategies were incorporated separately for caregivers and children.<br><br>For children: theme-based activities.<br><br>For parents/caregivers: videos to engage the children in media screen–free activities.<br><br>PLUMS will be implemented for 8 weeks and will consist of 8 themes.	In the caregivers' module, online videos will be disseminated. The program includes 1 introductory video and 7 small video sessions (5 min long) based upon the weekly theme. The objective of these videos are:<br><br>- To help the parents change the home media environment, provide alternatives to ST.<br>- To provide tips and cues for parenting skills, and engage the child in media-free activities of their choice.<br><br>These videos will also provide 10 alternative activities per week for children (the caregivers will be encouraged to reward the children for abiding by the rules and accomplishing goals).	The child module's lesson plans were drawn from educational disciplines like: skill building, motor coordination, learning, goal setting, music, dance, arts, and crafts.<br><br>This module contains activities for the child that need the caregiver's supervision. To capture the child's interest and channel their energy, specific activities have been selected. This module emphasizes observational learning and increasing the attention span of the child.<br><br>The authors provide following resources as an alternative to ST for the children:<br>- A reading book called "Chika Chika Boom Boom".<br>- Pretested and self-developed comic book on the consequences of ST.<br>- Coloring book with crayons.
Family@play	DURATION/ORGANIZACION	SESSIONS FOCUS	SESIONES GRUPALES
	Participant families in the intervention group attended six, one-hour group sessions each week. Five of the sessions were targeted at both parents, with the sixth session focusing specifically on the role fathers play in their young children's lives.	Sessions are focused on:<br><br>a) Non-selectively decreasing ED use, for instance by use of budgeting and removing ED options from children's bedrooms;<br>b) Selectively reducing ED use by setting rules/ boundaries around use, and limiting participation based on context (for instance, only when child is not eating)<br>c) Displacing ED use with other activities such as looking at books or being	Group sessions discussed the family-based activities to be undertaken by families and used an anticipatory guidance perspective to facilitate group-based problem solving to possible challenges.<br><br>Each session included goal setting specific to each family's circumstances and requirements. SMS messaging was used between group sessions to support adherence to goals: each participant received a personalised SMS encouraging them to achieve their previously-stated goal for the week.<br><br>The key messages of the program which were reinforced throughout the intervention were:<br><br>- Increase knowledge about ED recommendations and outcomes of ED use.<br>- Increase awareness and implementation of strategies to participate in healthy levels of ED use.<br>- Teach families how to practice behaviour modification such as planning and monitoring.
Case manager support	PROTOCOL	CORE ELEMENTS OF THE INTERVENTION	COMMUNICATION IN FOUR DOMAINS

<table>
<tr><th>PROGRAM</th><th colspan="6">INTERVENTION MAIN IDEAS</th></tr>
<tr><td></td><td colspan="2">1. A survey was completed that elicited three components of social cognitive theory: outcome expectations, self-efficacy, and volitional control.

2. Over the subsequent 4 months, the case managers kept in touch with the families by monthly phone and e-mail contact. Newsletters (with short articles about the target behavior, tips for development, relevant cartoons) were also mailed to all participants every month, with content tailored to the study group.</td><td>Estuvieron en contacto con las familias para formarles en base a ciertos elementos principales:

• Information about the targeted behavior change and its effects on health and development outcomes.
• Theories of behavior change used in the intervention.
• Training in successful communication, notably active listening.
• Concrete goal-setting
• Information about the health behavior and its effects.
Cheerleading the parent's efforts</td><td colspan="3">Each of these subsequent contacts constituted ongoing communication in four domains:

1. The positive or negative effects of TV on child health and development (outcome expectations).
2. Encouragement to the mother in building confidence to modify the child's TV viewing (intervention on self-efficacy).
3. Strategies for modifying the child's TV viewing (intervention on volitional control).
Assessment and counselling in the parent's stage of change for modifying TV viewing.</td></tr>
<tr><td rowspan="2">Intervention for reducing screen time</td><th colspan="4">INTERVENTION COMPONENTS</th><th colspan="2">MATERIALS</th></tr>
<tr><td colspan="4">The participants in the study group were exposed to the four intervention components at two week intervals

- First intervention component: In order to reduce ST, parents were asked to read age-appropriate books to their children daily; a family mealtime with TV turned off was advised; children were encouraged to offer alternative activities to watching TV, such as reading books; the parents were supported to place ‘no TV or screen’ signs on each TV or screen at home. Alternative ways of spending time was offered when not sitting in front of a screen. Then the parents were supported to remove their child’s TV or computer from his or her bedroom.
- Second intervention component: it was a counselling call. This call encouraged families to make their home screen free; it provided the benefits of a screen-free home, and difficulties to establish and keep a screen-free home.
- The third intervention component: included a picture book showing a family while making their home screen-free. It gave data about increasing conversation among family members, decreasing children’s ST and consequences of increased ST, such as violence.
- The fourth intervention component: included information about stories of families that were able to decrease their ST.</td><td colspan="2">The intervention consisted of three printed materials and interactive CD’s and one counselling call, intending to decrease ST.

- The first set of printed materials was given after the baseline questionnaire, followed by a counselling phone call two weeks later.
- The second and third printed materials were distributed at the fourth and sixth week. A follow-up questionnaire was done eight weeks after the start of the study. The printed materials and CD’s were aimed to decrease ST at home. CD recordings included harmful effects of TV, video and computer games and a list of alternative activities to watching TV and other screens.</td></tr>
<tr><td rowspan="2">parent-focused intervention</td><th>PROTOCOL</th><th colspan="4">THE INTEGRATED APPROACH</th><th>DYADIC APPROACH</th></tr>
<tr><td>- Four assessments at baseline
- Six weeks (mid-term)</td><td colspan="4">The intervention incorporated several key components. Participants received biweekly educational materials via the WhatsApp, with each set of materials requiring approximately 20 min to review. The materials included:</td><td>Employed the same intervention contents as the integrated approach, with the exception that the intervention materials were limited to PA and SB (including sedentary ST).</td></tr>
</table>

<table>
<tr><th>PROGRAM</th><th colspan="3">INTERVENTION MAIN IDEAS</th></tr>
<tr><td></td><td>- 12 weeks (pos-tintervention)
24 weeks (follow-up).</td><td>- Individual reports on their children’s current behaviours
- The gaps between their current behaviours and recommended guidelines
- Knowledge about the health benefits of improving behaviours
- Examples to improve behaviours,
- Strategies for goal setting and habit development,
- Potential barriers and strategies.
Additionally, three 30-minute workshops were delivered through Zoom meetings.</td><td></td></tr>
<tr><td rowspan="2">INTERVENTION TO REDUCE CHILDREN’S TELEVISION VIEWING</td><td>GENERAL INFORMATION</td><td colspan="2">ACTIVITIES AFTER SCHOOL</td></tr>
<tr><td>Each of the 7 sessions included components for the child, the day care or preschool provider, and the parents. The weekly 20-minute interactive, educational sessions were led by program staff, but day care and preschool staff were actively encouraged to participate.</td><td colspan="2">- To reinforce the program goals, additional materials and suggested classroom activities were provided to day care and preschool staff.
- After each class, materials and activities were sent home with each child to foster discussion between parents and children.
- Children were also encouraged to ask their parents for help in completing these activities at home.
- Day care and preschool staff and parents were provided with the list of alternative activities generated by the children and the brochure Television and the Family by the American Academy of Pediatrics.
- The children also made “no television” signs, which were sent home with a weekly calendar and “no television” stickers with instructions for parents to monitor and reward children by placing a sticker on the calendar every day the child refrained from watching TV or video shows.
- Materials from the National TV Turnoff Week Internet site were also used.</td></tr>
<tr><td rowspan="3">behavioral interventions to improve habits</td><td>GENERAL INFORMATION</td><td>TEACHERS TRAINING</td><td>INTERVENTION
Pilot Intervention</td></tr>
<tr><td rowspan="2">The intervention was based in Social Cognitive Theory, and was developed after conducting a series of focus groups with parents, teachers, and school administrators, direct observation of environments in the preschools, and surveys. The activities of the intervention were focused on 3 principal goals:
1) Drinking water instead of sugar-sweetened beverages.
2) Eating fruits and vegetables at snack times
3) Engaging in PA rather than ST during free time.</td><td rowspan="2">The activities centered around a story line of 4 fictional characters: Anita and Julián, both preschool-aged children, a turtle named George, and a hummingbird named Moti. Teachers underwent training before the intervention and received additional monthly training for 8 months for the PI and 6 months for the EI. Training not only consisted of how to deliver the intervention but also included training on nutrition and PA appropriate for preschoolers, such as age-appropriate serving sizes, making healthy choices, food hygiene, and identifying and preventing eating difficulties. Research staff visited preschools weekly to ensure that the intervention was being implemented appropriately.</td><td>The PI consisted of incorporating new activities into the existing school curriculum, such as “motor stories” where children heard about different activities in a story, such as growing and eating fruits and vegetables, and after the story the children participated in these activities.

These daily activities focused on underscoring the 3 principal goals of the intervention in a fun way, using puppets, replica models of foods, pre-recorded songs, and pop-up books. One hour per day was dedicated to specific motor activities that were part of the intervention, which included games and activities where children had some kind of structured group PA.

Classrooms were also provided with personal water bottles for the children and an organizer for them.

To encourage behavior change, teachers were also given a board displaying the children’s names and traffic light stickers to indicate the adequacy of children’s observed drinking, eating, and PA habits throughout the day. This board was displayed in the classroom and was used as a means of encouraging children to strive to develop good habits, with care not to single out any particular student.</td></tr>
<tr><td>Enhanced Intervention</td></tr>
</table>

<table>
<tr><th>PROGRAM</th><th colspan="4">INTERVENTION MAIN IDEAS</th></tr>
<tr><td></td><td></td><td></td><td colspan="2">In addition to the to the content of the Pilot intervention, the Enhanced Intervention included activities for children to do with their parents; these were a continuation of school-based activities. For example, in school, children learned how to play certain games that included PA, and then parents were shown how to continue these activities at home. Parents were trained by teachers in workshops, and information was also provided in a workbook. In the workbook, parents provided evidence of completion of a given activity, and for each activity completed, children and their parents were given a different magnet for their refrigerator with images of the theme characters engaging in healthy activities.</td></tr>
<tr><td rowspan="2">“Movement-to-music video program”</td><td colspan="4">GENERAL INFORMATION OF PARENTS` AND CHILDREN`S FORMATION</td></tr>
<tr><td colspan="4">Further, the mothers and children in the intervention group were instructed to use the movement-to-music video program DVD every other day from the beginning of week two to the end of week eight. The movement-to-music video program consisted of three separate exercise programs, each lasting 10 minutes. As per the instructions, the videos could be used individually or consecutively in order to allow the mother and child to choose the suitable amount of exercise for themselves.</td></tr>
<tr><td rowspan="2">Parental Education for limiting screen time in early childhood</td><td colspan="3">PARENTS` PRESENTIAL FORMATION</td><td>PARENTS` TELEPHONICALL FORMATION</td></tr>
<tr><td colspan="3">Parents in the Educational group received 30 minutes of in-person active counselling with pre-designed content targeted at reduction of ST in a language the caregivers could understand. They were guided to incorporate:

- Age-appropriate responsive parenting skills
- Increase interactive play of the infant, limit screen exposure
- Modify parental media habits.

The content was delivered as a one-to-one structured talk, and a printed pamphlet with these instructions was also handed over to the primary caregiver at the end of the session.</td><td>The same was also reinforced telephonically on monthly basis (5 sessions) till the end of the study i.e., or 6 months from enrolment.</td></tr>
<tr><td rowspan="3">Intervention modeled around previously published screen-time interventions</td><td colspan="3">PARENTS IN THE INTERVENTION GROUP</td><td rowspan="2">PARENTS IN THE INTERVENTION AND CONTROL GROUPS</td></tr>
<tr><td>GENERAL INFORMATION</td><td colspan="2">TYPE OF INFORMATION PROVIDED</td></tr>
<tr><td>Parents in the intervention group received a 10-minute behavioral counselling by trained study personnel directly after the health maintenance visit, which included information on the health impact of ST in children and provided strategies to decrease ST. These strategies included suggestions such as:

- Removing the TV from the child’s bedroom.
- Encouraging meals to be eaten without the TV on.
- Budgeting of the child’s ST.</td><td colspan="2">- Families were encouraged to try a 1-week TV turn off, in which children were encouraged to spend time without the TV and were provided with a calendar and stickers to reward the children for days without the TV.
- Contingency planning for time spent not watching TV was promoted.
- Activities for the child, during this session, included providing a story to parents about TV viewing (The Berenstain Bears and Too Much TV) and creating a list of non-television related activities.

The intervention group also received a Canadian Pediatric Society handout titled “Promoting Good TV Habits.”</td><td>Parents of children in both the intervention and control groups received standardized counselling from trained study personnel on safe media use, which included information about:
- TV rating systems
- Internet safety
- Limiting exposure to violent programming.

They both received a previously published Canadian Pediatric Society parent handout titled “Managing Media in the Home.”</td></tr>
</table>

<table>
<tr><th>PROGRAM</th><th colspan="3">INTERVENTION MAIN IDEAS</th></tr>
<tr><td rowspan="2">Developmental pediatrics clinic setting counseling</td><td>GENERAL INFORMATION</td><td colspan="2">INTERVENTIONS</td></tr>
<tr><td>ST interventions are classified according to their intensity as low and high intensity</td><td colspan="2">High intensity interventions are those having at least three interactions with family/child or the presence of an ED to limit ST. The intervention in the study is high intensity as it included three consecutive counselling sessions in a developmental behavioral paediatric clinic. In each session, at least one parent received about 10-15 minutes behavioral counselling intervention face to face by a developmental paediatrician after the medical visit. The counselling included the AAP recommendations about screen viewing, information on the health and developmental effects ST on children, strategies to reduce ST (reading books or playing together) and an explanation about the detrimental effects of background TV exposure</td></tr>
<tr><td rowspan="2">Parents' Screen Time Reduction for Preschool Children Program (PSTRPP)</td><td colspan="3">GENERAL INFORMATION</td></tr>
<tr><td colspan="3">This program comprises three sessions, three hours long, over two weeks. The three sessions include:

- An instructive session, providing recommendations and suggestions regarding the child's ST
- A restrictive session; rules being set to reduce the child's ST and parental modelling.
- Parent-child interaction, including alternative activities for children to decrease their ST.
Each session had activities based on the Theory of Planned Behaviors to promote parents' positive attitudes, increase parents' subjective norms, and increase parents' perceived control, behaviors and parents' intentions on their behaviors concerning decreasing their child's ST.</td></tr>
<tr><td rowspan="3">F5K TV reduction program's</td><td rowspan="2">GENERAL INFORMATION</td><td colspan="2">INTERVENTION</td></tr>
<tr><td>IN CLASS</td><td>AT HOME</td></tr>
<tr><td>The culturally adapted F5K TV reduction curriculum, which was taught by the same study staff member (SJ) directly to the children in the classroom setting. Because the F5K curriculum was originally designed and tested among a mostly non-Latino white sample of preschoolers, a cultural adaptation process was undertaken to increase intervention efficacy and relevance for this underserved, disadvantaged population, as recommended by an expert panel. The cultural adaptation process consisted of three phases:

- Qualitative interviews with parents to inform the curriculum
- Forward- and back-translation of F5K with decentring, in which the source text itself could be modified if there were problematic phrases or to increase cultural sensitivity.
- A "practice" trial of the curriculum in two Latino Head Start classrooms, which were not involved in the study, in which staff both taught the curriculum (SJ) and observed and noted lesson components that needed improvement or that were well received.</td><td>F5K was taught over 7–8 weeks and consisted of seven themes (see appendix Table 1 of the article), each composed of five to six lesson plans of 15–30 minutes organized around the theme (see appendix Figure 1 of the article). The lesson plan were drawn from four educational disciplines:

1. Language Arts.
2. Math.
3. Music and Movement.
4. Arts and Crafts</td><td>F5K also included a parent newsletter sent home weekly to inform parents of the lessons and to provide optional home activities for the parents to complete with their preschoolers. For parents of limited Spanish or English proficiency, the directions and goals of the take-home materials were provided to them verbally by study staff when they picked up their children from the Head Start centre.</td></tr>
</table>

<table>
<tr><th>PROGRAM</th><th colspan="4">INTERVENTION MAIN IDEAS</th></tr>
<tr><td rowspan="3">Primary Care Weight Management Program</td><td>BEFORE THE INTERVENTION</td><td colspan="3">DURING THE INTERVENTION</td></tr>
<tr><td>INVESTIGATORS TRAINING</td><td>COMPONENTS</td><td>RECOMMENDATIONS DURING SESSIONS</td><td>RECOMMENDATIONS BY MESSAGES</td></tr>
<tr><td>Prior to the intervention, physicians from treatment sites participated in a training seminar from study investigators to improve the content and delivery of their interactions with families regarding healthy lifestyle choices. The training included methods for evaluating and discussing obesity-related lifestyle behaviors with families of preschool-aged children, including healthy nutrition, PA, sleep, and SB. Physicians were also instructed regarding appropriate goal-setting strategies, including a brief discussion of motivational interviewing techniques.</td><td>The study intervention included two primary components:

1. Physician-family health behaviour conversations during well-child visits.
2. Four monthly visits with a RDN (registered dietitian nutritionist) to evaluate, educate, and implement improved feeding habits and nutritional choices.

A third optional component of the intervention included counselling sessions with a social worker to help families overcome barriers to change, such as food security, family relationships, and general parenting strategies (i.e., authoritative vs. authoritarian, permissive, or uninvolved). Families were informed of the option to meet with a social worker for a behavioural counselling visit, and those interested were scheduled for a separate appointment with the social worker at the paediatric office. Visits lasted approximately one hour.</td><td>Behavioural counselling visits were designed to help enable families to incorporate the behaviour changes recommended by the RDNs during their sessions. Parents were counselled regarding authoritative parenting practices, such as how to be warm and nurturing while also providing firm and consistent expectations and opportunities for independence.
For example, parents were encouraged to establish rules regarding meal times and food choices, while also being sensitive to the child's satiety cues and supportive when the child tried new foods.</td><td>Health behaviour education focused on the “Healthy Counts” messaging, which includes nine health behaviours:
1. >8 h of sleep
2. 7 breakfasts/week.
3. 6 home cooked meals/week.
4. 5 servings of fruit and vegetables/day.
5. 4 positive self-messages/day.
6. 3 servings of low fat dairy/day.
7. 2 h or less of ST/day.
8. 1 h or more of PA/day.
9. 0 sugar-sweetened beverages/day)

Other covered topics included division of responsibility with feeding:
- self-regulation,
- MyPlate
- meal planning,
- healthy snacks,
- daily nutrient needs,
- picky eating,
- goal setting
- Any other specific nutritional concerns.

A team of registered dietitians, paediatricians, and childhood-obesity researchers created educational handouts tailored to each well-child visit from 2–5 years of age and each of the four RDN visits, which were distributed by physicians during well-child visits and RDNs during the appropriate appointments</td></tr>
<tr><td rowspan="2">Stop and Play</td><td>GENERAL INFORMATION</td><td colspan="3">INTERVENTION MODULE</td></tr>
<tr><td>It incorporates the 6 constructs under SCT, namely, knowledge, goal setting, self-efficacy, outcome expectation, observational learning, and problem-solving to improve parental knowledge and skills in reducing ST among</td><td colspan="3">The intervention module was developed through a process of consultation with a panel of health experts. To ensure its local cultural suitability, it was first pretested among 35 mother-child dyads of preschool children, who were not part of the main study. The delivery of the module was performed by the primary researcher, a physician. The intervention module took 4 weeks to be completed. Overall, 3 videos relevant to the educational content were created using whiteboard animation, and an additional video that portrayed success stories from other</td></tr>
</table>

PROGRAM	INTERVENTION MAIN IDEAS	
	preschool children, by providing feasible activities that are culturally tailored as alternatives. The *Stop and Play* intervention also promoted self-directed play, a potential solution for parents who tend to use ST as *digital babysitters*.	mothers was also included. All videos were uploaded to YouTube and set to restricted viewing to avoid public access. The links to the YouTube videos were shared on a weekly basis with the participating mothers in the intervention group, with each video being approximately 3 minutes in duration.<br><br>The videos were complemented with 2 infographic materials to reinforce the educational content. During the fourth week of intervention, there was a 12-hour problem-solving session, whereby the mothers could discuss the challenges they faced with the researcher via a private WhatsApp chat. On the basis of the viewing data on the YouTube channel, there was an average of 1 to 1.5 views per person per video. In addition, more than half of the participants in the intervention group (102/180, 56.7%) interacted with the researcher during the WhatsApp problem-solving session to obtain further assistance in reducing their child's ST. An average of 3 interactions per participant was recorded for this session. The total time taken to complete the intervention, including viewing the weekly videos and reviewing the infographics, was approximately 60 minutes over 4 weeks. The intervention group also received a starter pack consisting of a program introduction, a *Stop and Play* fridge magnet, and a screen media diary for parents to report their child's ST.

Note = AAP: American Academy of Paediatrics; BMI: Body mass index; CON: control; ED: electronic device; F5K: Fit 5 Kids; FNPA: Family Nutrition and Physical Activity; GEE: Generalized estimating equation; INT: intervention; PA: physical activity; PLUMS: Program to lower unwanted media screen; PSTRPP: Parents' Screen Time Reduction for Preschool Children Program; SB: Sedentary behaviour; ST: screen time; TV: television.
